# Novel alleles of the *VERNALIZATION1* genes in wheat are associated with modulation of DNA curvature and flexibility in the promoter region

**DOI:** 10.1186/s12870-015-0691-2

**Published:** 2016-01-27

**Authors:** Alexandr Muterko, Ruslan Kalendar, Elena Salina

**Affiliations:** Laboratory of Plant Molecular Genetics and Cytogenetics, The Federal Research Center Institute of Cytology and Genetics, Lavrentyeva Avenue 10, Novosibirsk, 630090 Russian Federation; Department of Common and Molecular Genetics, Plant Breeding and Genetics Institute – National Center of Seed and Cultivar Investigation, Ovidiopolskaya Road 3, Odessa, 65036 Ukraine; Laboratory of Plant Genomics and Bioinformatics, RSE “National Center for Biotechnology”, Sh. Valikhanov 13/1, Astana, 010000 Kazakhstan; University of Helsinki, Institute of Biotechnology, MTT Plant Genomics Laboratory, Biocentre 3, P.O. Box 65, Viikinkaari 1, Helsinki, 00014 Finland

**Keywords:** Cereal, Genetic variation, Flowering time, Vernalization requirement, Wheat, DNA curvature, VRN-box, *VRN1* genes, Promoter, New alleles, Anomalous migration

## Abstract

**Background:**

In wheat, the vernalization requirement is mainly controlled by the *VRN* genes. Different species of hexaploid and tetraploid wheat are widely used as genetic source for new mutant variants and alleles for fundamental investigations and practical breeding programs. In this study, *VRN-A1* and *VRN-B1* were analysed for 178 accessions representing six tetraploid wheat species (*Triticum dicoccoides*, *T. dicoccum*, *T. turgidum*, *T. polonicum*, *T. carthlicum*, *T. durum*) and five hexaploid species (*T. compactum*, *T. sphaerococcum*, *T. spelta*, *T. macha*, *T. vavilovii*).

**Results:**

Novel allelic variants in the promoter region of *VRN-A1* and *VRN-B1* were identified based on the change in curvature and flexibility of the DNA molecules. The new variants of *VRN-A1* (designated as *Vrn-A1a.2, Vrn-A1b.2 – Vrn-A1b.6* and *Vrn-A1i*) were found to be widely distributed in hexaploid and tetraploid wheat, and in fact were predominant over the known *VRN-A1* alleles. The greatest diversity of the new variants of *VRN-B1* (designated as *VRN-B1.f*, *VRN-B1.s* and *VRN*-*B1.m*) was found in the tetraploid and some hexaploid wheat species.

For the first time, minor differences within the sequence motif known as the VRN-box of *VRN1* were correlated with wheat growth habit. Thus, *vrn-A1b.3* and *vrn-A1b.4* were revealed in winter wheat in contrast to *Vrn-A1b.2*, *Vrn-A1b.5, Vrn-A1b.6* and *Vrn-A1i*. It was found that single nucleotide mutation in the VRN-box can influence the vernalization requirement and growth habit of wheat. Our data suggest that both the A-tract and C-rich segment within the VRN-box contribute to its functionality, and provide a new view of the hypothesised role of the VRN-box in regulating transcription of the *VRN1* genes. Specifically, it is proposed that combination of mutations in this region can modulate vernalization sensitivity and flowering time of wheat.

**Conclusions:**

New allelic variants of the *VRN-A1* and *VRN-B1* genes were identified in hexaploid and tetraploid wheat. Mutations in A-tract and C-rich segments within the VRN-box of *VRN-A1* are associated with modulation of the vernalization requirement and flowering time. New allelic variants will be useful in fundamental investigations into the regulation of *VRN1* expression, and provide a valuable genetic resource for practical breeding of wheat.

**Electronic supplementary material:**

The online version of this article (doi:10.1186/s12870-015-0691-2) contains supplementary material, which is available to authorized users.

## Background

One of the key factors governing the adaptability of wheat to a wide range of environmental conditions is allelic diversity within genes regulating seasonal growth habit (vernalization requirement) and photoperiod response. The vernalization requirement determines the need for a prolonged exposure to low temperature in order for plants to transition to the reproductive phase of development. According to their response to vernalization, wheat plants can be categorised as winter (strong vernalization sensitivity), spring (not sensitive to vernalization), or facultative types (intermediate growth habit). The vernalization requirement and growth habit in wheat is largely controlled by four major loci, which are also critical in determining flowering and maturity times: these include *VRN1* (a MADS-box transcription factor), *VRN2* (a zinc-finger CCT domain gene - *ZCCT*), *VRN3* (homologous to the Arabidopsis gene *FLOWERING LOCUS T*) and *VRN4* (MADS-box transcription factor) [1–4].

The *VRN* genes interact epistatically (reviewed by [5]). The products of *VRN2* expression downregulate transcription of *VRN3* [3] through competitive interaction with other CCT-domain proteins. This occurs in conjunction with the replacement of the HAP2 (HEME ACTIVATOR PROTEIN2) subunit of the HAP2/3/5 complex, which binds with the *VRN3* promoter [6]. In turn, VRN3 proteins are transported from the leaves to the shoot apical meristem [7] where they interact with transcription factor FDL2 (FLOWERING LOCUS D-like2) resulting in the formation of a protein complex that activates expression of the *VRN1* genes [3, 8] and likely its paralogs *FUL2* (*FRUITFULL 2*) and *FUL3* [9]. During vernalization, *VRN2* transcription is progressively downregulated by the vernalization conditions (short photoperiod and low temperature) and increasing activation of *VRN1*, which indirectly suppresses *VRN2* [2, 10]. It is supposed that subsequent expression of *VRN1* in leaves is important for maintaining the repression of *VRN2* transcription in winter wheat after vernalization [9]. Interaction between *VRN1*, *VRN2* and *VRN3* form a regulatory positive feedback loop [5, 10] in which *VRN3* is the integrator of the vernalization and photoperiod floral pathways [3, 11]. A change in expression of any of the *VRN* genes in this feedback loop results in modulation of the transcription profiles of the other two genes and a change in flowering time. It is assumed that *VRN4* is active in the leaves and operates upstream (or is a part) of the *VRN1/VRN2/VRN3* feedback loop, herewith *VRN1* is the earliest target (direct or indirect) of *VRN-D4* among these three genes [4].

The *VRN2* locus includes three tandemly duplicated *ZCCT* genes. The recessive *vrn2* allele is associated with a loss of function for all copies of the *ZCCT* genes from all homologous genomes which can be caused by missense mutations within the CCT domain coding region [2, 12] or the presence of null alleles [13]. Likely, this is the main reason why accessions carrying recessive *vrn2* have so far been described only for *T. monococcum* [2] and have not been observed in varieties of polyploid wheat [5]. Natural variation for *VRN3* has been found only in the B genome [3, 14]. The dominant *Vrn-D4* allele is less widespread in wheat, although it is frequent in hexaploid wheat from India and nearby regions [15, 16], and is mainly predominant in accessions of *T. sphaerococcum* [4]. Thus, the vernalization requirement of an overwhelming number of spring accessions of hexaploid and tetraploid wheat is determined by the allelic diversity of *VRN1* genes.

The *VRN1* genes have been mapped to the middle of the long arms of the chromosomes 5 homeologous group [1]. Allelic variants at the *VRN1* genes are largely responsible for the division of wheat into winter, spring, and facultative types. The presence of the dominant *VRN-A1* alleles results in complete elimination of the vernalization requirement (spring type), whereas the presence of either *VRN-B1* or *VRN-D1* alleles alone is associated with some residual vernalization response and later flowering (facultative type) [17, 18]. *VRN1* genes possess no less than two regulatory regions, located in the promoter and first intron respectively [19, 20]. Among dominant alleles of *VRN-A1* the predominant carry mutations within the promoter region (in particular the *Vrn-A1a*/*b*/*d*/*e* and *Vrn-A*^*m*^*1a*/*g* alleles), while the dominant alleles of *VRN-B1* (*Vrn-B1a*/*b*/*c*) and *VRN-D1* (*Vrn-D1a* and *Vrn-D1s* alleles) are mostly determined by mutations in the first intron [1, 10, 19–23]. However, a later study identified the dominant *VRN-B1* allele (hereafter designated as *Vrn-B1(ins)*), which has a retrotransposon insertion in the promoter and confers a spring growth habit. Still, this insertion was detected mainly for *T. carthlicum* (86 % accessions) and only in 3 accessions of *T. dicoccum* [24]. Similarly, a recent study identified a 174-bp insertion within the *VRN-D1* promoter region, which possibly promoted the basal activity level of the *VRN-D1* gene (*Vrn-D1c* allele) [25]. Furthermore, in accessions of *T. aestivum* were identified from 1 to 4 copies of the *VRN-A1* gene [26]. This copy number variation (CNV) polymorphism is an important trait, which influences on vernalization requirement duration and flowering time of wheat [27, 28].

The promoter region of the *VRN1* gene contains multiple regulatory sites [1, 29, 30] most important of which are the CArG-box, VRN-box and ACGT-motif. The CArG-box (a common binding site for MADS-box proteins) is located 180 bp upstream of the site of transcription initiation but downstream of the ACGT-motif [1]. However, during subsequent studies it was found that the vernalization requirement is preserved in plants where the CArG-box is fully deleted (*vrn-A*^*m*^*1b* allele) [31], indicating that the CArG-box is not critical for the determination of the vernalization requirement. Pidal et al. [31] analysed the 5' UTR of the *VRN-A1* alleles in spring and winter *T. monococcum* accessions carrying mutations within the promoter region of *VRN-A1*. This led to the identification of a sequence located upstream from the CArG-box, containing mutations which were associated with the spring growth habit. Furthermore, the same region is also altered in two dominant, spring *Vrn-A1a* and *Vrn-A1b* alleles found in polyploid wheat [19]. This sequence of 16 bp (TTAAAAACCCCTCCCC) was designated as the VRN-box. It is assumed that the VRN-box is involved in determination of the vernalization requirement. Located upstream from CArG- and VRN-boxes are the binding sites for bZIP transcription factors. Overall, within the entire 2.3 kb promoter region of *VRN1* several such DNA motifs were found, all with a ACGT core sequence. These consisted of a G-box (CACGTG) and four putative hybrid boxes: A/G, A/C, G/A, G/C, which are binding sites for TaFDL2 (orthologue of *FLOWERING LOCUS D-LIKE 2* in wheat) and form a protein complex with TaFT (VRN3) [8]. Thus, interaction of TaFDL2 with TaFT and with the bZIP-binding sites of the *VRN1* promoter forms the basis of a mechanism which provides regulation of the transcription of *VRN1* by *VRN3*. Furthermore, activation of *VRN1* expression by vernalization is associated with changes in histone methylation in different regions of the promoter [30] and intron-1 [32]. Additionally, recent reports have highlighted the post transcriptional regulation of *VRN1* expression involving sequences within the first intron [4, 33].

The less widespread species of hexaploid and tetraploid wheat often attract the attention of researchers as genetic sources of new mutant variants and alleles of agronomically valuable traits. For example, the tetraploid wheat species *T. dicoccoides*, *T. dicoccum* and *T. carthlicum*, and hexaploid wheat *T. spelta* and *T. compactum* were where the alleles *Vrn-A1d*, *Vrn-A1e* [19], *Vrn-B1(ins)* [24] and *Vrn-D1s* [23] respectively, were first identified. And although implementation of these alleles in wheat breeding is difficult to achieve within a short time span, nevertheless, new data provide significant benefits. This research serves to expand our knowledge about the structure and function of *VRN* genes, facilitates identification and accurate localization of regulatory sites and regions, and furthers our understanding of the ways and mechanisms determining the development of wheat, its vernalization requirement and flowering time.

In the present study the regulatory regions of the *VRN1* genes were analysed in tetraploid and hexaploid wheat species in order to evaluate genetic variation and identify of new allelic variants. The phenomenon of anomalously slow migration of DNA fragments in polyacrylamide gels which indicates presence of curved DNA was used as the first step in identification of differences in gene regulatory regions. Focusing our attention on investigation of the *VRN-A1* gene, we also analysed *VRN-B1* and generalized these data with the results of our previous research on *VRN-D1* in the same accessions of hexaploid wheat [23]. This has strengthened the connection between genotype and phenotype of experimental plants, and enabled us to predict the possible effects of new mutant variants in the regulatory regions. Novel variants of *VRN-A1* and *VRN-B1* identified herein can be useful not only for practical breeding, but also for fundamental investigation of the mechanisms regulating *VRN1* gene expression. In particular, new data can contribute to the more accurate localization of regulatory regions within the *VRN1* promoter and in the first intron.

## Results

### Identification of three variants of *Vrn-A1a*

**Fig. 1:**
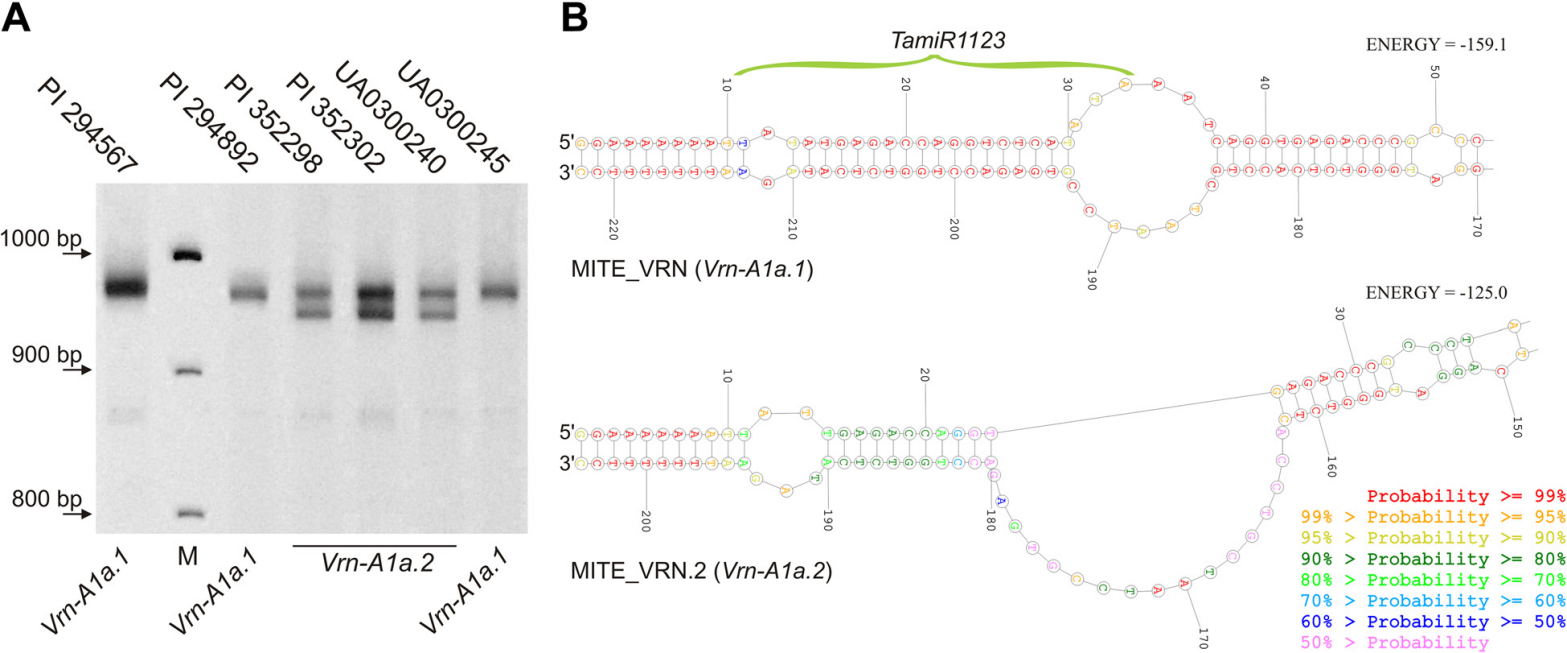
Identification and analysis of the *Vrn-A1a* allelic variants using PCR with primers VRN1AF/VRN1-INT1R. **a** Separation of promoter region PCR fragments of *Vrn-A1a* for *T. compactum* accessions in polyacrylamide gel (853 bp fragments show a very low amplification). M – DNA molecular size marker. **b** RNA secondary structures (partial) predicted for MITE_VRN and MITE_VRN.2 from *Vrn-A1a.1* and *Vrn-A1a.2* respectively. The localization of the hairpin loop structure, which is needed for processing of the *TamiR1123* microRNA is indicated

Polyacrylamide gel electrophoresis of PCR fragments of the *VRN-A1* promoter region for *T. compactum* and *T. dicoccum* accessions revealed three combinations of amplicons, representing three *Vrn-A1a* allelic variants. These variants were designated as *Vrn-A1a.1* (PCR fragments 853 and 944 bp), *Vrn-A1a.2* (fragments 853, 924 and 944 bp) and *Vrn-A1a.3* (fragments 765 and 853 bp). The *Vrn-A1a.1* and *Vrn-A1a.3* variants (also known as *Vrn-A1a*) were previously identified in hexaploid and tetraploid wheat respectively [19]. The new variant, *Vrn-A1a.2*, differs from *Vrn-A1a.1* by the amplification of an additional 924 bp fragment (Fig. 1a). This amplicon was cloned and sequenced (GenBank: KR782255 (*T. compactum*, PI 352302)). Subsequent analysis revealed a 16 bp deletion and 4 single nucleotide deletions within the MITE insertion when compared to *Vrn-A1a.1* (GenBank: AY616458). Yu et al. [34] recently reported that a 222 bp insertion of MITE_VRN in the promoter region of the *Vrn-A1a.1* allele processes a 23 bp sequence of microRNA (*TamiR1123*), which is complementary to the previously identified *miR507* [35]. The RNA secondary structure was reconstructed to determine the effect of a 16 bp deletion on the ability to form a stable hairpin loop, which is needed for microRNA processing (Fig. 1b). Results indicated that this deletion not only encompasses 30 % of the sequence, which encodes *TamiR1123*, but also prevents formation of the hairpin loop structure precluding further processing of microRNA. To investigate the possibility that MITE_VRN is read with the target gene as one transcript with an alternative promoter located within MITE, a similarity search of wheat ESTs using the MITE_VRN sequence was performed. As a result, 9 transcripts from 6 clusters (Ta.98996, Ta.153105, Ta.11542, Ta.127802, Ta.219174, Ta.125868) with identity no less than 82 % and query coverage > 77 % (of these, 6 accessions had coverage of MITE greater than 90 %) were identified. While for 7 accessions MITE was located within the mRNA, for two transcripts the similarity region began from start position of mRNA and correspond of 24 bp position of MITE sequence.

### Discovery the novel *Vrn-A1i* allele

**Fig. 2:**
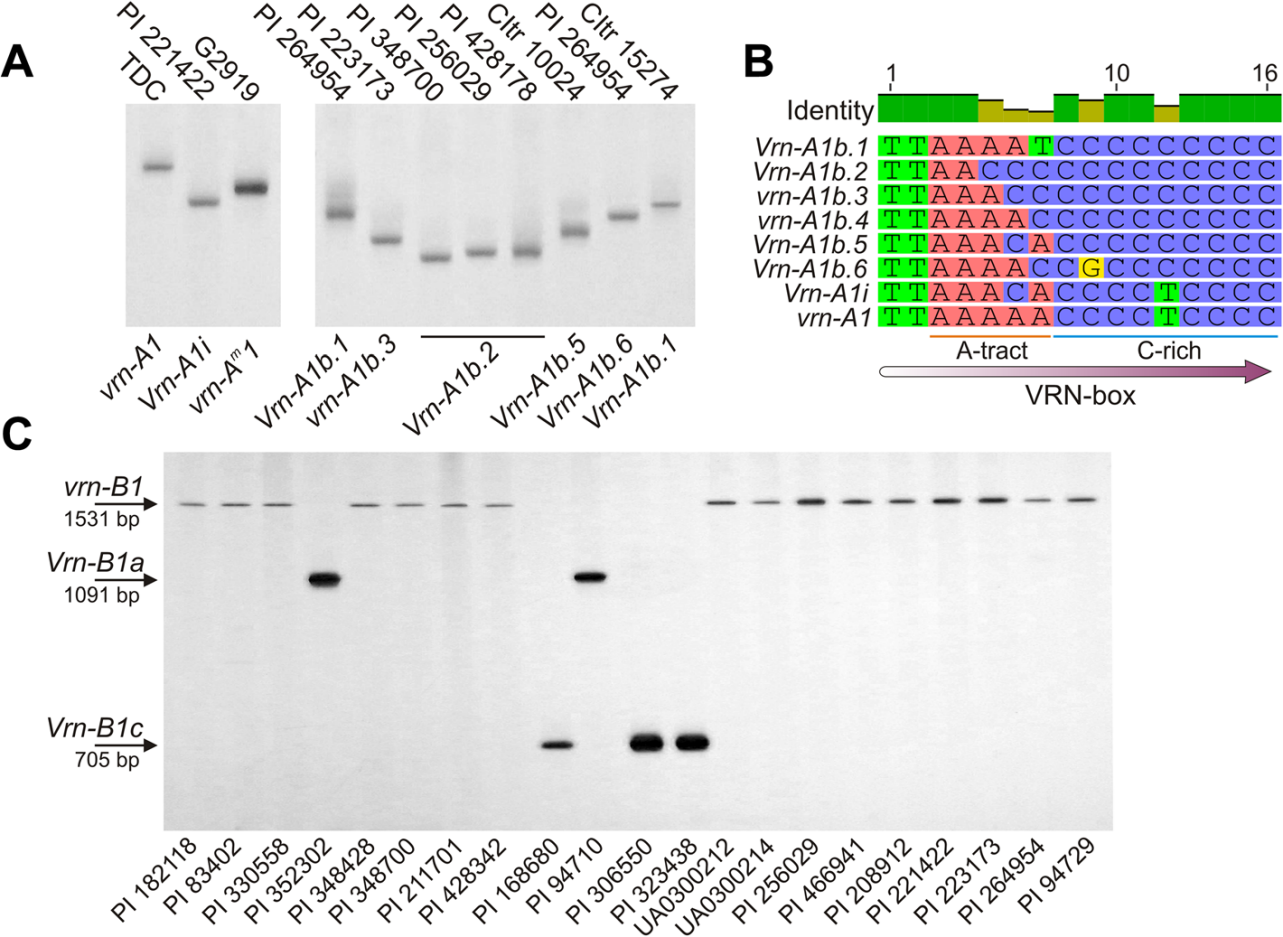
Identification of the *VRN-A1* and *VRN-B1* alleles during PAGE. **a** Discrimination of identified herein *Vrn-A1i* and variants of *Vrn-A1b* under electrophoresis optimized for low temperature conditions. PCR fragments of the *VRN-A1* promoter region were amplified using the primer pair VRN1AF/VRN1-INT1R. **b** Mutations within VRN-box differentiate *Vrn-A1i* and variants of *Vrn-A1b* from *vrn-A1* (GenBank: KM047646 (*Vrn-A1b.1*), KM047641 (*Vrn-A1b.2*), KM047647 (*vrn-A1b.3*), KM047651 (*vrn-A1b.4*), KM047652 (*Vrn-A1b.5*), KT692944 (*Vrn-A1b.6*), KM016790 (*Vrn-A1i*), AY747600 (*vrn-A1*)). The DNA sequence of the VRN-box of the *Vrn-A1b* variants as well as VRN-box of *Vrn-A1i* contains polymorphism within the A-taract, while the VRN-box of *Vrn-A1b.5* and *Vrn-A1b.6* differed from the VRN-box of *Vrn-A1i* and *vrn-A1b.4* respectively, by the SNP within the C-rich segment. **c** PCR analysis of the *VRN-B1* intron-1 for hexaploid and tetraploid wheat

Separation of PCR fragments of the *VRN-A1* promoter region, in PAA gels of those fragments identified in agarose gels as the recessive *vrn-A1* allele (713 bp), revealed fast migrating amplicons in some accessions of tetraploid wheat. These amplicons migrated closely with 705 bp fragments amplified for reference from the accession of *T. monococcum* with *vrn-A*^*m*^*1* (Fig. 2a). PCR fragments from two accessions of *T. turgidum* (PI 208912, PI 221422), one *T. durum* (PI 74830) and a reference accession of *T. monococcum* (PI 428170) were cloned and sequenced (GenBank: KM016789 (*T. monococcum*, PI 428170), KM016790 (*T. turgidum*, PI 208912), KM016791 (*T. durum*, PI 74830), KM016792 (*T. turgidum*, PI 221422)). Sequence analysis confirmed the identity of the *vrn-A*^*m*^*1* allele for the accession of *T. monococcum* and revealed a single nucleotide polymorphism within the A-tract (AnTm, n + m > =4) of the VRN-box for accessions of *T. turgidum* and *T. durum* (Fig. 2b). This new *VRN-A1* allelic variant was designated as *Vrn-A1i*, according to the nomenclature of vernalization response genes proposed by Yan et al. [19]. The curved DNA migrates more slowly than non-bent DNA of the same length during polyacrylamide gel electrophoresis, as has been demonstrated [36–38]. Usually the anomalously slow electrophoretic mobility is associated with increasing curvature of the DNA molecule, determined by the A-tract bending [39, 40]. The adenine to cytosine mutation leads to a break of the A-tract (A3 instead of A5) within the VRN-box of *Vrn-A1i.* Furthermore, replacement of adenine with cytosine inside the A-tract is accompanied by an increase in flexibility of the DNA segment through reduction of the rigid local bend, due to the shorter A-tract, and presence of two more flexible nucleotide steps “AC” and “CA” instead of the rigid “AA” steps. Distribution analysis of local bending angles and curvature of the DNA molecule (Additional file 1: Figure S1) shows that the changes in the A-tract result in straightening of DNA fragments, which gives the *Vrn-A1i* amplicons a characteristic shape. This leads to increasing migration in PAA gels of the *Vrn-A1i* PCR fragments compared with amplicons of *vrn-A1*, which migrate anomalously slow as a result of their curvature, as determined by the intact A-tract within the VRN-box.

### Discovery of new variants of the *Vrn-A1b* allele

The next important result obtained during resolution of *VRN-A1* promoter region amplicons in PAA gels was the detection of a difference in migration rate for PCR fragments with length near to the DNA marker of *Vrn-A1b* (Fig. 2a). This was not observed during agarose gel separation or in non-optimized or denaturized PAGE. Several polymorphic amplicons were cloned and sequenced (GenBank: KM047641 (*T. dicoccoides*, PI 233288), KM047642 (*T. dicoccoides*, PI 256029), KM047643 (*T. dicoccoides*, PI 352322), KM047644 (*T. spelta*, PI 330558), KM047645 (*T. spelta*, PI 348700), KM047646 (*T. turgidum*, PI 264954), KM047647 (*T. dicoccum*, UA0300214), KM047648 (*T. durum*, PI 655432), KM047649 (*T. vavilovii*, PI 428342), KM047650 (*T. turgidum*, PI 223173), KM047651 (*T. dicoccoides*, PI 466941), KM047652 (*T. dicoccum*, UA0300212), KT692944 (*T. durum*, CItr 10024), KT692945 (*T. turgidum*, PI 220356)). The result of multiple alignments with the reference sequence of the dominant *Vrn-A1b* allele (GenBank: AY616461) enabled the identification of six variants of *Vrn-A1b*. All contain three common features in common with *Vrn-A1b*: a 20 bp deletion located 137 bp upstream of the start codon, the “T” deletion upstream of the VRN-box and a “T- > C” replacement within the C-rich segment of the VRN-box. Five variants (*Vrn-A1b.1-b.5*) differ from each other by a polymorphism of the A-tract within the VRN-box. These variants were designated as *Vrn-A1b.1* (A-tract = “AAAAT”), *Vrn-A1b.2* (A-tract mutated to “AACCC”), *vrn-A1b.3* (A-tract mutated to “AAACC”), *vrn-A1b.4* (A-tract mutated to “AAAAC”) and *Vrn-A1b.5* (A-tract mutated to “AAACA”), while *Vrn-A1b.6* carries an additional “C- > G” transversion within the C-rich segment compared to *vrn-A1b.4* (Fig. 2b). In this nomenclature the *Vrn-A1b.1* corresponds to the original *Vrn-A1b* allele (GenBank: AY616461) previously identified by Yan et al. [19]. It should be noted that PCR fragments of the *vrn-A1b.3* and *Vrn-A1b.5* as well as *vrn-A1b.4* and *Vrn-A1b.6* variants migrate in PAA gels at a similar rate, indicating the importance of the length of the A-tract of the VRN-box in generating small observable differences in migration rate. Overall, “A- > C” transversions within the A-tract were accompanied by a reduction in the length of the A tract and curvature of DNA molecules that resulted in a decrease in the anomalously slow migration of PCR fragments of the *Vrn-A1b* variants during PAGE.

### Distribution of the *VRN-A1* alleles in wheat

*VRN-A1* alleles with mutations in the promoter region and alleles carrying large deletions in the first intron are distributed unevenly among different wheat species. In particular, 74 % carry alleles with mutations in the promoter region, whereas only 26 % carry the 7.2 kb deletion in the first intron (*Vrn-A1c (Langdon)* allele). No mutations in intron-1 were detected in any hexaploid species or in wild and domesticated emmer wheat, *T. dicoccoides* and *T. dicoccum*. The highest distribution of *Vrn-A1c* was recorded in *T. durum* (71 %) and *T. polonicum* (58 %), and *Vrn-A1c* was detected only one accession within subsets of *T. carthlicum* and *T. turgidum*. The dominant *Vrn-A1a* allele was found in 6 accessions of *T. compactum* and 4 accessions of *T. dicoccum*. In each of these species two variants of *Vrn-A1a* were identified. *Vrn-A1a.1* and the newly- identified *Vrn-A1a.2* were detected in *T. compactum* accessions, while *Vrn-A1a.1* and *Vrn-A1a.3* were recorded in *T.dicoccum*. The novel *Vrn-A1i* allele was identified in 5 accessions of *T. turgidum* and in one sample of *T. durum*.

The variants of *Vrn-A1b* are widespread in tetraploid and hexaploid wheat species: 80 and 77 % from alleles carrying mutations in promoter for tetraploid and hexaploid wheat respectively (Additional file 2: Table S1, Additional file 3: Table S2). The different variants of *Vrn-A1b* were represented in all accessions of *T. spelta* and *T. dicoccoides* with a mutant *VRN-A1* promoter. Among all five variants of the *Vrn-A1b* allele, the canonical variant *Vrn-A1b.1* was observed only in 8 % cases. Most (92 %) carried the variants described herein - *Vrn-A1b.2-b.6*. In tetraploid wheat *Vrn-A1b.2* was found in all spring accessions of *T. dicoccoides* and in 42 % accessions of *T. dicoccum*. The *vrn-A1b.3* allele predominates in *T. turgidum* (40 % accessions). *Vrn-A1b.6* was identified in all accessions of *T. polonicum* with *Vrn-A1b* and in samples of *T. durum* and *T.turgidum.* The original *Vrn-A1b* allele (*Vrn-A1b.1* variant) was found only in two accessions of *T. durum* and 3 accessions of *T. turgidum*. In hexaploid wheat new variants of *Vrn-A1b* are represented in almost all analysed species with exception of *T. sphaerococcum*. The greatest diversity in *Vrn-A1b* was detected in *T. spelta*, where the *Vrn-A1b.2*, *vrn-A1b.3* and *Vrn-A1b.6* variants were identified*.* Two accessions of *T. compactum* (PI 262666 and PI 41023) possessed both *vrn-A1* and *Vrn-A1b.6* alleles.

The dominant allele *Vrn-A1e*, previously identified in *T. dicoccum* [19], was detected in two accessions of *T. carthlicum* and one accession of *T. dicoccum*. PCR fragments of the *Vrn-A1e* and *Vrn-A1f* alleles have approximately the same length of 658 bp. Hence, *Vrn-A1e* was confirmed by sequencing (GenBank: KT361213). Moreover, predicted DNA curvature for the PCR fragments of *Vrn-A1f* and *Vrn-A1e* allows us to assume that fragments of *Vrn-A1f* will migrate slower in PAA gels relative to *Vrn-A1e* due to the presence of two additional bends generated by the A-tract within the VRN-box and T-tract within CArG-box. Finally, *Vrn-A1f* likely occurs in wheat of the *Timopheevii* section [29].

Interestingly, the new variants of *VRN-A1* identified here using changes in gel retardation (following changes in curvature and flexibility of DNA molecules) are more common in wheat species than the previously known *VRN-A1* alleles. This indicates that probably these new variants are actively involved in natural selection as useful adaptations to environmental conditions.

### Discovery of new *VRN-B1* variants

**Fig. 3:**
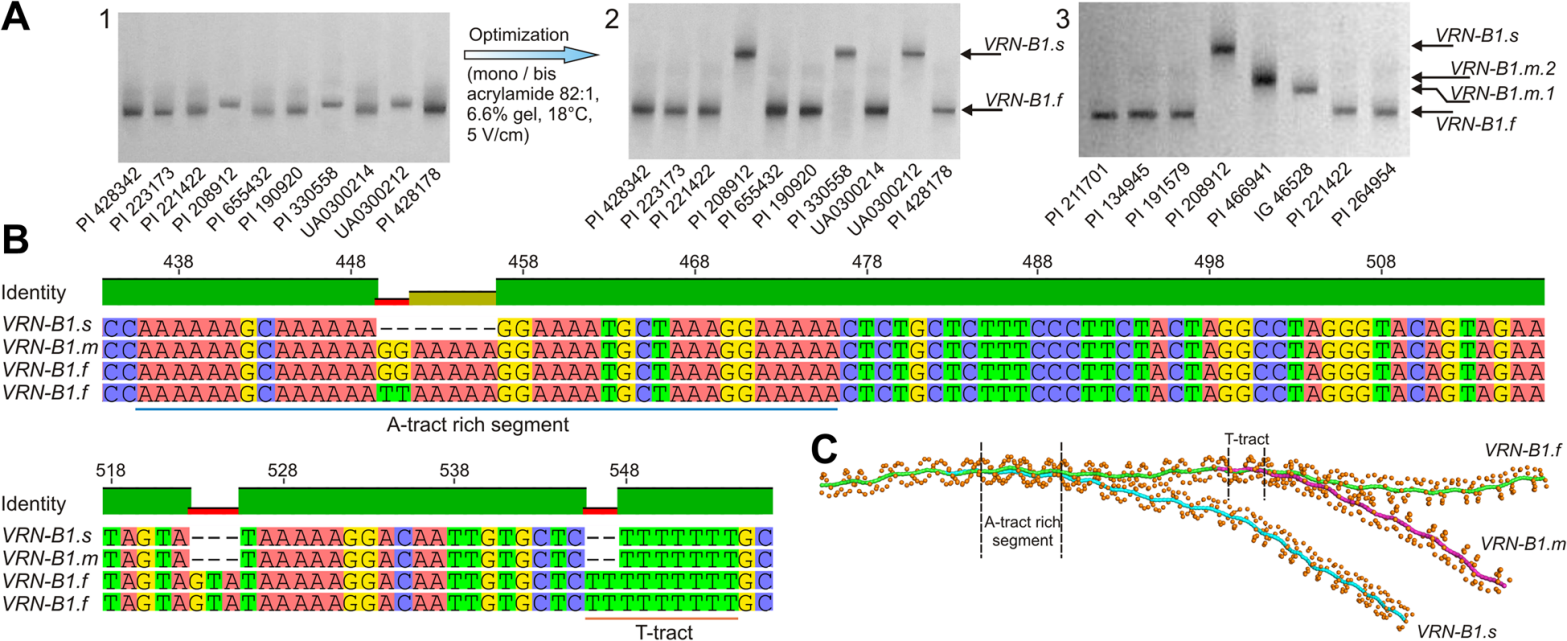
Mutations within the promoter of *VRN-B1* indicate tree sequence variants. **a** Identification of the *VRN-B1* variants during PCR with primer pair Pr1/Pr2. Separation of PCR fragments under conditions of low conformational dynamics of DNA molecules contribute to the clear discrimination of the alternative promoter variants. 1 – non-optimized PAA electrophoresis at room temperature without cooling, in 10 % gel, mono/bis-acrylamide ratio 29:1, under 5 V/cm; 2 – the same amplicons resolved in PAGE optimized for low temperature conditions; 3 – support for the contribution of localized DNA bends as determined by the 7 bp deletion within the A-tract rich segment in anomalously migrating PCR fragments of *VRN-B1.s*, the 7 bp deletion distinguishes *VRN-B1.s* from *VRN-B1.m*, observed splitting of *VRN-B1.m* on two variants. **b** Comparison of DNA sequences encompassing the center of PCR fragments detecting *VRN-B1* variants (GenBank: KR782252 (*VRN-B1.s*), KT361212 (*VRN-B1.m.2*), KR782253 (*VRN-B1.f* from *T. turgidum*), KR782254 (*VRN-B1.f* from *T. polonicum*)). Region showed includes three deletions in *VRN-B1.s*, two located within the A-tract rich segment and T-tract cause local bends and lead to increased curvature of the DNA molecule. Positions are indicated relative to the start of the *VRN-B1.f* PCR fragment. **c** Superposition of predicted DNA paths of PCR fragments of the *VRN-B1* promoter variants. Sites of local DNA bends caused by the 7 and 2 bp deletions within the A-tract rich segment and T-tract are indicated

Results of *VRN-B1* promoter region analysis found that almost all accessions of *T. carthlicum* (with the exception of PI 168672) carry a retrotransposon insertion (*Vrn-B1(ins)* allele). Under certain electrophoresis conditions the difference in the migration rate of PCR fragments with the intact *VRN-B1* promoter was revealed (Fig. 3a). These variants were designated as *VRN-B1.f*, *VRN-B1.m* and *VRN-B1.s* (according to the migration rate of relevant PCR fragments: “f” = fast, “m” = medium, “s” = slow). The difference in migration rates between amplicons that enabled the detection of alternative *VRN-B1* promoter variants was observed only by polyacrylamide gel separation and increased with reduced conformational dynamics and greater stability of DNA molecules (Fig. 3a).

For a more detailed investigation, the amplicons of *VRN-B1.f*, *VRN-B1.s* and *VRN-B1.m* were cloned and sequenced (GenBank: KR782252 (*T. turgidum*, PI 208912), KR782253 (*T. turgidum*, PI 191579), KR782254 (*T. polonicum*, PI 134945), KT361212 (*T. dicoccoides*, PI 466941)). DNA sequence alignment of these alternative PCR fragments identified 8 SNPs (T102C, G174A, A297C, T341G, +360C, +370C, C558T, C729G) and three short deletions at 7 bp (“GGAAAAA”), 3 bp (“GTA”) and 2 bp (“TT”) for *VRN-B1.s* relative to *VRN-B1.f*, where *VRN-B1.f* corresponds to the *vrn-B1* allele with an intact promoter. *VRN-B1.m* differs from *VRN-B1.s* by the absence of 7 bp deletion. Furthermore, it was found that SNPs C654G and A693G, identified in the related DNA sequence of *VRN-B1.f*, are associated with *VRN-B1.m*. Although altogether the mutations of *VRN-B1.s* result in a reduction in amplicons length by just 10 bp, the *VRN-B1.s* PCR fragments (total length 958 bp) migrate through PAA gels much slower than amplicons of *VRN-B1.f* (968 bp). The difference, seen during PAGE with conditions optimized for low temperature, appeared greater than 100 bp when measured relative to the DNA molecular weight marker. Similarly, amplicons of *VRN-B1.m* (965 bp) migrate faster than amplicons of *VRN-B1.s* (958 bp), but slower than *VRN-B1.f* (968 bp). The 7 bp and 2 bp deletions were located within A-tracts, which in turn were located near the center of the fragment (Fig. 3b). Both of these factors contribute to a change in DNA curvature, leading to the observed anomalously slow migration of *VRN-B1.s* and *VRN-B1.m* PCR fragments in PAA gels compare to *VRN-B1.f* amplicons (Fig. 3c). The 2 bp deletion reduces the length of the T-tract from 9 to 7 bp. The more distal 7 bp deletion located within the A-tract rich segment encompasses a −549 to −509 bp region counting from the start codon of the native *VRN-B1* (*VRN-B1.f*). This region includes 6 A-tracts, which are separated by dinucleotide steps (“GC” and “GG”). The polymorphism of a dinucleotide step separating the 2-nd and 3-rd A-tracts for *VRN-B1.f* accessions was detected. Comparison of this region within 15 accessions available from GenBank showed that *T. dicoccoides* and *T. turgidum* include the dinucleotide “GG”, while *T. aestivum*, *T. durum*, *T. polonicum* and *T. carthlicum* carry “TT” (Fig. 3b).

To determine what contribution the 7 bp deletion within the A-tract rich segment makes to the change of global DNA curvature and observed retardation of the *VRN-B1.s* amplicons in PAA gels, the PCR products of accessions PI 208912 (*VRN-B1.s*), PI 466941 (*VRN-B1.m*), IG 46528 (the sequence of which (GenBank: KM586665) corresponds to *VRN-B1.m*) and PI 191579 (*VRN-B1.f*) were analysed in same electrophoretic conditions (Fig. 3a). The results showed that the contribution of the 7 bp deletion in anomalous migration of the *VRN-B1.s* PCR fragments is less significant predominant (5−10 %) than the contribution of a 2 bp reduction in the T-tract. Thus both these contributions are equally important to electrophoretic retardation of the *VRN-B1.s* amplicons. Additionally, it was found that amplicons from IG 46528 (*VRN-B1.m.1*) show no significant difference in migration compared to amplicons from PI 466941 (*VRN-B1.m.2*), despite both accessions carrying *VRN-B1.m*. Sequence alignment showed that PCR fragment of *VRN-B1.m.1* contains “T” deletion at position 230 bp and G341T transversion eliminating of the A-tract (“ATG” instead “ATT”), that explains observed difference in migration rate between amplicons of *VRN-B1.m.1* and *VRN-B1.m.2,* where *VRN-B1.m.1* migrates faster than *VRN-B1.m.2*.

### Distribution of *VRN-B1* alleles

Distribution analysis of the *VRN-B1* variants showed that *VRN-B1.f* is widespread in winter and spring accessions of hexaploid and tetraploid wheat species, while *VRN-B1.s* is identified predominantly in accessions which, according to passport or experimental date, have a spring growth habit. Distribution of variants *VRN-B1* in the different hexaploid wheat species is unequal. *VRN-B1.s* is found only in accessions of *T. compactum* and *T. spelta*. A sharp decrease in *VRN-B1* promoter variant diversity, likely the result of a genetic bottleneck and founder effects due to speciation, is observed for such species as *T. macha*, *T. vavilovii* and *T. shaerococcum*, where all analysed accessions were characterized by the *VRN-B1.f*. Conversely, in tetraploid wheat all species (except *T. carthlicum* containing a TE insertion in *VRN-B1* promoter) were characterized by the more uniform distribution of *VRN-B1* variants with *VRN-B1.f* predominant. *VRN-B1.m* was identified in only two accessions of *T. dicoccoides* (PI 352322, PI 466941). Interestingly, all accessions of *T. polonicum* with *Vrn-A1c* are associated with *VRN-B1.s*, while accessions carrying *Vrn-A1b* are characterized by the *VRN-B1.f*. Similarly, *VRN-B1.s* was found in accessions of *T. spelta* with *Vrn-A1b.2*.

Multiplex PCR was used to investigate *VRN-B1* intron-1. The forward primer Ex1/C/F was previously designed to amplify *Vrn-A1c* [20]. However, sequence alignment indicated that this primer anneals to *VRN1* genes from all three genomes (A, B and D). Accordingly, the primer combination Ex1/C/F, Intr1/B/R3 and Intr1/B/R4 was optimised for investigation of *VRN-B1*. This new multiplex PCR provides four DNA-markers in PCR fragments with lengths of 1531, 1091, 1055 and 705 bp, to discriminate *vrn-B1*, *Vrn-B1a*, *Vrn-B1b* and *Vrn-B1c* alleles respectively (Fig. 2c). The dominant alleles of *Vrn-B1* with mutations in intron-1, prevailed in hexaploid wheat species. In particular, the dominant *Vrn-B1a*, *Vrn-B1b* or *Vrn-B1c* alleles were not detected in tetraploid wheat *T. turgidum*, *T. polonicum*, *T. carthlicum*. Only one accession *T. durum* carries *Vrn-B1a*, while 2 and 5 accessions in *T. dicoccum* and *T. dicoccoides* respectively carry *Vrn-B1c*. In hexaploid wheat, the *Vrn-B1c* was identified in *T. spelta* (33 %), *T. macha* (one accession) and *T. sphaerococcum* (one accession), while *Vrn-B1a* was detected only in *T. compactum* (50 %).

### Polymorphism of the VRN-box is associated with vernalization requirement and flowering time

According to passport data, almost all accessions with *vrn-A1b.3* have a winter growth habit with the exception of two accessions of *T. spelta* (PI 306550, PI 323438). Nevertheless, DNA-marker analysis has shown that the vernalization insensitive phenotype of these spring accessions of *T. spelta* is determined by the dominant *Vrn-B1c* allele. Some of the accessions with *vrn-A1b.3* were grown in a glasshouse without vernalization under a long photoperiod (>16 h) and were then genotyped by *VRN* markers (Tables 1 and 2). The accessions PI 428342, PI 655432 and UA0300214 (detected only *vrn-A1b.3*, *VRN-B1.f*) failed to reach stem elongation 120 days (4 months) after planting, when the experiment was terminated. Only PI 223173 (*vrn-A1b.3*, *VRN-B1.s*) showed a later flowering time (128 days). This confirms that accessions with *vrn-A1b.3* are characterized by a winter growth habit and retain the vernalization requirement. Similarly, the accession of *T. dicoccoides* (PI 466941) with *vrn-A1b.4* according to passport data has a winter growth habit and this was confirmed here experimentally (failed stem elongation after 120 days). In contrast, all accessions with *Vrn-A1b.2* and *Vrn-A1b.5* according to passport data have a spring growth habit which was also confirmed during the experimental glasshouse planting carried out in the present study. Nevertheless, almost all accessions with *Vrn-A1b.2* have *VRN-B1.s* and/or carry dominant alleles of the other *VRN1* genes (*VRN-B1/D1*). The accessions PI 428178 (*T. macha*) and PI 190920 (*T. dicoccum*) were only exceptions.

**Table 1:** PCR primers details

Primers	Primer sequence (5'-3')	Primer design	Annealing temp. °C	Amplified region	Allelic variant	PCR product size (bp)
VRN1AF	gaaaggaaaaattctgctcg	Yan et al. [19]	60	*VRN-A1* promoter	*vrn-A1*	713
VRN1-INT1R	gcaggaaatcgaaatcgaag				*Vrn-A1a.1*	853, 944
					*Vrn-A1a.2*	853, 924, 944
					*Vrn-A1a.3*	765,853
					*vrn-A* ^*m*^ *1*	705
					*Vrn-A1b*	691
					*Vrn-A1d*	685
					*Vrn-A* ^*m*^ *1g*	681
					*Vrn-A* ^*m*^ *1a*	671
					*Vrn-A1e*	659
					*Vrn-A1f*	658
					*vrn-A* ^*m*^ *1b*	656
Vrn-A1-intr_F	ccgtcgaaaggatcgctactg	This study	60	*VRN-A1* intron-1	*vrn-A1*	541
Vrn-A1-intr_R1	cttgtccccgtgagctacttac		60			
Ex1/C/F	gttctccaccgagtcatggt	Fu et al. [20]	58	*VRN-A1* intron-1	*Vrn-A1c (Langdon)*	522
Intr1/A/R3	aagtaagacaacacgaatgtgaga		58		*Vrn-A1c (IL369)*	2188
Pr1	tacccctgctaccagtgcct	Shcherban et al. [22]	60	*VRN-B1* promoter	*VRN-B1.f*	968
Pr2	ggccaaccctacaccccaag		60		*VRN-B1.s*	958
					*VRN-B1.m*	965
Pr1	tacccctgctaccagtgcct	Shcherban et al. [22]	60	*VRN-B1* promoter	*Vrn-B1(ins)*	1256
PrB/Ins/r2	tgcggtacgggttatccag	This study	60			
Ex1/C/F	gttctccaccgagtcatggt	Fu et al. [20]	61	*VRN-B1* intron-1	*Vrn-B1a*	1091
Intr1/B/R3	ctcatgccaaaaattgaagatga		61		*Vrn-B1b*	1055
					*Vrn-B1c*	705
Ex1/C/F	gttctccaccgagtcatggt	Fu et al. [20]	61	*VRN-B1* intron-1	*vrn-B1*	1531
Intr1/B/R4	caaatgaaaaggaatgagagca		61			
FT-B-INS-F	cataatgccaagccggtgagtac	Yan et al. [3]	63	*VRN-B3* promoter	*Vrn-B3a*	1765
VRN4-B-NOINS-R	ctatccctaccggccattag		63			
VRN4-B-NOINS-F2	gctgtgtgatcttgctctcc	Yan et al. [3]	63	*VRN-B3* promoter	*vrn-B3*	691
VRN4-B-NOINS-R	ctatccctaccggccattag		63		*vrn-B3b*	1581

**Table 2:** Genotype and days to flowering (DTF) of hexaploid and tetraploid wheat accessions grown >120 days under 16 h photoperiod without vernalization

Species	ID	*VRN* genotype	DTF (±2 days)
*T. sphaerococcum*	PI 182118	*vrn-A1/vrn-B1 (VRN-B1.f)/vrn-D1/vrn-B3*	53
	PI 83402	*vrn-A1/vrn-B1 (VRN-B1.f)/Vrn-D1a/vrn-B3*	72
*T. spelta*	PI 168680	*vrn-A1b.3/Vrn-B1c (VRN-B1.f)/vrn-D1/vrn-B3*	67
	PI 330558	*Vrn-A1b.2/vrn-B1 (VRN-B1.s)/Vrn-D1s/vrn-B3*	59
	PI 348428	*Vrn-A1b.2/vrn-B1 (VRN-B1.s)/Vrn-D1s/vrn-B3*	61
	PI 348700	*Vrn-A1b.2/vrn-B1 (VRN-B1.s)/Vrn-D1s/vrn-B3*	60
*T. compactum*	PI 211701	*vrn-A1/vrn-B1 (VRN-B1.f)/Vrn-D1s/vrn-B3*	77
*T. vavilovii*	PI 428342	*vrn-A1b.3/vrn-B1 (VRN-B1.f)/vrn-D1/vrn-B3*	-
*T. dicoccum*	UA0300212	*Vrn-A1b.5/vrn-B1 (VRN-B1.s)/vrn-B3*	46
	UA0300214	*vrn-A1b.3/vrn-B1 (VRN-B1.f)/vrn-B3*	-
*T. dicoccoides*	PI 256029	*Vrn-A1b.2/vrn-B1 (VRN-B1.s)/vrn-B3*	66^a^
	PI 466941	*vrn-A1b.4/vrn-B1 (VRN-B1.m.2)/vrn-B3*	-
*T. turgidum*	PI 208912	*Vrn-A1i/vrn-B1 (VRN-B1.s)/vrn-B3*	77
	PI 221422	*Vrn-A1i/vrn-B1 (VRN-B1.f)/vrn-B3*	102
	PI 223173	*vrn-A1b.3/vrn-B1 (VRN-B1.s)/vrn-B3*	128
	PI 264954	*Vrn-A1b.1/vrn-B1 (VRN-B1.f)/vrn-B3*	68
*T. durum*	PI 94729	*Vrn-A1c/vrn-B1 (VRN-B1.f)/vrn-B3*	54
	PI 74830	*Vrn-A1i/vrn-B1 (VRN-B1.s)/vrn-B3*	76
	PI 655432	*vrn-A1b.3/vrn-B1 (VRN-B1.f)/vrn-B3*	-
*T. carthlicum*	PI 532505	*vrn-A1/Vrn-B1(ins)/vrn-B3*	61

^a^PI 256029 with *Vrn-A1b.2* was reach stem elongation 7 days earlier than PI 264954 with *Vrn-A1b.1*

Two accessions of tetraploid wheat with *Vrn-A1i* (PI 208912 and PI 74830), according to passport data, have a spring growth habit, while four others were characterized by a winter growth habit. Nevertheless, in growing these “spring” and “winter” (PI 221422) accessions, all of them were able to flower without vernalization (Table 2), however flowering was significantly later than accessions with dominant *VRN-A1* or *VRN-B1* alleles. It should be noted that the “winter” accession (PI 221422) flowered 25 days later than “spring” (PI 208912, PI 74830), herewith they all have recessive *vrn-B1* and *vrn-B3* alleles. It is assumed that the decreased vernalization sensitivity and facultative growth habit is typical of accessions possessing *Vrn-A1i*.

The *Vrn-A1b.5* accession (UA0300212) flowered 22 days early than *Vrn-A1b.1* (PI 264954). Similarly PI 208912 and PI 74830 flowered 25 days earlier than PI 221422, while all of them carried *Vrn-A1i* (Table 2). The *Vrn-A1b.5* has the same polymorphism of the A-tract within VRN-box as *Vrn-A1i*, but it also contains common mutations characterizing all variants of the *Vrn-A1b* allele. Interestingly, in all of these cases the earlier accessions (UA0300212, PI 208912, PI 74830) carried *VRN-B1.s*. Moreover, the *VRN-B1.s* was identified in the PI 223173 accession, which later flowered without vernalization, in contrast to other accessions also carrying *vrn-A1b.3* and *VRN-B1.f*. This suggests an association between *VRN-B1.s*, a decrease of vernalization sensitivity and early flowering. It should be noted that the accession of *T. dicoccoides* (PI 466941) with *vrn-A1b.4* and *VRN-B1.m* failed to achieve stem elongation before the experiment was terminated, in contrast to accession PI 223173 with *vrn-A1b.3* and *VRN-B1.s*. Furthermore, it was found that accession UA0300212 (*Vrn-A1b.5*, *VRN-B1.s*) flowered 30 days earlier than PI 208912 and PI 74830 (*Vrn-A1i*, *VRN-B1.s*). This difference approximately corresponds to the difference between PI 264954 (*Vrn-A1b.1*, *VRN-B1.f*) and PI 221422 (*Vrn-A1i*, *VRN-B1.f*), which was 34 days (Table 2). In both cases, the compared accessions do not carry the known dominant *VRN-B1* and *VRN-B3* alleles, they have the same promoter variants of *VRN-B1* and differ only at the *VRN-A1* alleles with a common feature: “T- > C” SNP within the C-rich region of the VRN-box (Fig. 2b).

## Discussion

### Detection of the identified *VRN-A1* and *VRN-B1* alleles

The difference in curvature and flexibility of DNA molecules caused by the mutations within the VRN-box of *VRN-A1* allelic variants first described herein, or by deletions within A-tracts for promoter variants of *VRN-B1*, leads to modulation in the rate of anomalously migrating PCR fragments. More than 25 years ago it was shown that anomalous migration of intrinsically bent DNA molecules in PAA gels allows the detection of small sequence-specific DNA structure variations [41]. In our previous study the haplotypes of *VRN-D1* and *Ppd-A1* haplogroups were discriminated based on anomalous migration [23, 42]. In the present study we found that the sensitivity of PCR fragments migration rate in PAA gels to curvature of DNA molecules provides a good basis for the development of a simple and fast method to discriminate between the new *VRN-A1* and *VRN-B1* allelic variants. Nevertheless, identification of the new *VRN-A1* and *VRN-B1* variants requires very specific conditions of PAGE. Hence, the electrophoretic conditions were optimized to obtain the minimal loss of resolution during electrophoresis run under low and room temperature. For example, as seen in Fig. 3 during a typical neutral PAGE the difference between *VRN-B1* variants is insignificant or fully absent, whereas optimization of PAGE results in a clear discrimination between the two. Overall, the retardation of anomalously slow migrating PCR fragments and sharper bands definition are especially important for identification of the *Vrn-A1b* variants, where *Vrn-A1b.1*/*Vrn-A1b.6* and *vrn-A1b.3*/*Vrn-A1b.2* show a small difference in migration rate, and also are the predominant variants of *VRN-B1*. Adherence to recommendations proposed herein promotes the clear discrimination of *Vrn-A1b* and *VRN-B1* variants and prevents potential errors in the analysis of genetic material for vernalization experiments. For example, electrophoresis via agarose gels or PAA gels under standard conditions does not allow for the detection of differences between variants of *Vrn-A1b*. Thus, *vrn-A1b.3* and *vrn-A1b.4*, associated with vernalization requirement and winter growth habit can be erroneously taken as the known dominant *Vrn-A1b* allele, which is associated with a spring growth habit.

### Polymorphism of the *VRN-B1* promoter region results in a change of curvature of the DNA molecule

Analysis of the *VRN-B1* promoter region identified three sequence variants (*VRN-B1.f, VRN-B1.s* and *VRN-B1.m*), which could be resolved during separation in PAA gels under low conformational dynamics of DNA molecules. Compared to *VRN-B1.f* (968 bp), the *VRN-B1.s* (958 bp) contains three short deletions at 2, 3 and 7 bp, while *VRN-B1.m* (965 bp) differs from *VRN-B1.s* by the absence of a 7 bp deletion. Nevertheless, *VRN-B1.s* migrates slower than *VRN-B1.m* which is in turn slower than *VRN-B1.f*. As was described previously the curved DNA molecules migrate slower in polyacrylamide but not in agarose gels [36, 41] at a rate mostly determined by the length and localization of A-tracts [38–40]. Anomalously slow migration of the PCR fragments of *VRN-B1.s* and *VRN-B1.m* is determined by the localization of 2 and 7 bp deletions within A-tracts. In particular, a 2 bp deletion reduces the length of the 9 bp T-tract. As it was previously shown by Koo et al. [39] and our recent study on discrimination of haplotypes of *VRN-D1* [23], with decreasing length of the A-tract DNA curvature increases, while an A-tract length of more than 7 nt, reduces curvature through antiphase compensation of equivalent bends in opposite directions. Hence, a reduction in length of the T-tract from 9 to 7 nucleotides leads to an increase in local curvature of this DNA segment. This explains the contribution of the 2 bp deletion to the anomalous slow migration of *VRN-B1.s* and *VRN-B1.m*. The 7 bp deletion is located within A-tract rich segment, which includes 6 A-tracts separated by dinucleotide steps. Thus, a bend produced by the 1st tract is compensated by the bend of the 2nd tract, which is oriented in the opposite direction. Similarly, the 4th tract compensates for the bend of the 3rd tract, and the 6th tract for the 5th. As a result, curvature in this region is insignificant. However in *VRN-B1.s* the 4th tract is not compensated by an opposite bend of the 3rd tract due to the absence, through the 7 bp deletion encompassing this segment. This leads to increased curvature of the DNA molecule and retardation of *VRN-B1.s* amplicons in the PAA gel. The effect of the change of local curvature on the slowdown of *VRN-B1.s* amplicons is significantly enhanced due to both DNA bends, caused by the 7 and 2 bp deletions, localized the center of the fragment. In a previous study, Shcherban et al. [43] noted variability in the *VRN-B1* promoter region for some accessions of *T. dicoccoides* including 7, 3 and 2 bp deletions, which carry as designated here the *VRN-B1.m* and *VRN-B1.s* variants. However, the present study is the first to examine the distribution of these *VRN-B1* variants among wheat species and their probable phenotypic association, and to describe a method for their detection. The combination of the dominant *VRN-A1* alleles with *VRN-B1.s* was associated with early flowering (Table 2). Although it seems unlikely that mutations characterizing of *VRN-B1.s* have an effect on regulatory sites since they are located a long distance away from the transcription start site, these mutations could exert an influence through a distal enhancer or silencer. Also, it cannot be ruled out that the significant bending of the DNA molecule inside the *VRN-B1.s* promoter region, confirmed here by anomalously slow migration in PAA gels, facilitates an interaction between a remote enhancer and regulatory sites within the *VRN1* promoter; thus activating transcription via DNA looping. Additionally, an insertion in the promoter region of *Vrn-D1c*, associated with increasing transcript levels, is located more than 60 bp upstream of the A-tract rich region and 600 bp upstream of the transcription start site [25]. Nevertheless, while the association between promoter sequence variants of *VRN-B1* and phenotypic traits requires further investigations these variants are highly valuable as polymorphic, codominant DNA markers in genetic analysis.

### Study of *VRN-B1* intron-1

A number of marker systems have already been proposed for identification of the dominant *VRN-B1* alleles carrying large deletions within intron-1 [20, 44]. The multiplex PCR system optimized in the present study provides DNA markers almost 150 bp shorter than the existing analogues to discriminate between the intact intron-1 and the three dominant *Vrn-B1a, Vrn-B1b* and *Vrn-B1c* alleles. Furthermore, the use of the forward primer Ex1/C/F simultaneously in the two separate PCR assays for identification of alleles of the *VRN-B1* gene and dominant allele *Vrn-A1c* [20] is economically advantageous. Distribution analysis of the *VRN-B1* dominant alleles, showed that the *Vrn-B1c* allele is more commonly found in spring accessions of hexaploid and tetraploid wheat species than *Vrn-B1a* or *Vrn-B1b* (which was not detected), while an insertion retrotransposon in the promoter region of the *VRN-B1* gene is likely specific to tetraploid wheat *T. carthlicum*, where it is the main determinant of spring growth habit (Additional file 3: Table S2). The majority of the spring wheat germplasm surveyed here carried the dominant *VRN-B1* alleles in combination with dominant *VRN-A1*. Interestingly, accessions of *T. compactum* with dominant *VRN-B1* are characterized by the *Vrn-B1a* allele, while some accessions of *T. spelta* carry *Vrn-B1c* exclusively.

### Less frequent *VRN* alleles in hexaploid and tetraploid wheat

All accessions of *T. sphaerococcum* carry the recessive *vrn-A1* allele (with intact of *VRN-A1* promoter and intron-1) and according to passport data, 16 accessions have a spring growth habit and two samples have a facultative or winter growth habit. However, *Vrn-B1c* was identified in only one accession and *Vrn-D1a* in just two [23]. Thus, the spring growth habit of the predominant number of *T. sphaerococcum* accessions is mostly determined by genes non-allelic to *VRN1* or *VRN-B3*. This is consistent with the assumption that the spring growth habit of *T. sphaerococcum* accessions is largely controlled by the dominant *Vrn-D4* allele [4].

### The newly described allele *Vrn-A1a.2* can indicate the presence of an additional copy of the *VRN-A1*

Yan et al. [19] identified two variants of *Vrn-A1a,* named here *Vrn-A1a.1* and *Vrn-A1a.3*. They assumed that *Vrn-A1a.1* is specific to hexaploid wheat, while *Vrn-A1a.3* is specific to tetraploid wheat. In the present study *Vrn-A1a.3* was detected only in tetraploid wheat, whereas *Vrn-A1a.1* was identified in both hexaploid and tetraploid wheat species. A new allele, *Vrn-A1a.2*, first identified here was detected only in accessions of hexaploid wheat.

Southern blot hybridization using a probe matched to the *VRN-A1* region between exons 4 and 8 [19] showed that isogenic lines carrying *Vrn-A1a.1* and *Vrn-A1a.3* (TDD and Anza respectively) have the same hybridization fragments with a similar intensity as lines possessing the recessive *vrn-A1* allele (TDC, Winter-Anza). These data indicate that accessions with *Vrn-A1a* have the same copy number of *VRN-A1* genes as accessions with *vrn-A1*, such as the TDC line. Recently, using a *VRN1* copy number Taqman assay, it was found that TDC contains one copy of *VRN-A1* [4]. Identification in the *Vrn-A1a.2* of additional copy of the promoter region and exon-1, comparing with *Vrn-A1a.1* and *Vrn-A1a.3* allows us to assume that accessions with *Vrn-A1a.2* contain no less than 2 copies of *VRN-A1* (i.e. one copy more than *Vrn-A1a.1* and *Vrn-A1a.3*), differing from each other by the 16 bp deletion during MITE_VRN insertion in the promoter region.

The original MITE from the *Vrn-A1a.1* allele, when transcribed to RNA forms a highly stable hairpin loop structure (Fig. 1b) that facilitates later processing to form a microRNA - *TamiR1123* [34]. However the mutant variant of MITE identified in this study (designated Spring.2 or MITE_VRN.2) from the *Vrn-A1a.2* allele has a 16 bp deletion encompassing 8 bp within the DNA sequence of *TamiR1123* which leads to a change in RNA secondary structure preventing the loop in the pri-miRNA (Fig. 1b). The 16 bp deletion is flanked by a nucleotide repeat (“CAGGT” in this case) that is characteristic of deletions within *VRN1* genes [45] and suggests that the doublestranded DNA break repair mechanism of non-homologous end-joining, mediated by the single-strand annealing-like mechanism [46] can be invoked to explain their emergence. Features of sequence and target site duplication characterize MITE_VRN as a Mutator-like element (MULE) [34]. It is known that MULEs can harbor promoters of genes for transcription [47]. Furthermore, most miRNA genes in plants contain the TATA box motif and are transcribed by RNA polymerase II [48, 49]. In any case, this promoter must be located upstream of the sequence encoding *TamiR1123* since the transcription level of *TamiR1123* positively correlates with the transcription level of *Vrn-A1a.1* in the TDD line [34]. Hence, the *Vrn-A1a.2* promoter must be intact, since the 16 bp deletion within MITE is located downstream of the start position of *TamiR1123*. This is true if *TamiR1123* and *Vrn-A1a* are transcribed as a single product [34]. Results of the BLAST analysis of wheat ESTs assume that mRNA can be read with an alternative promoter located within MITE upstream of the 24 bp site, indicating that MITE_VRN and the target gene in reality can be read as a single transcript. The possibility of direct or indirect induction of *Vrn-A1a* by *TamiR1123* to explain the positive correlation in their transcript levels seems unlikely since *Vrn-A1a* is not the only source of *TamiR1123* - it is also present in winter accessions [34].

### Polymorphism of the VRN-box is associated with modulation of the vernalization requirement and flowering time of wheat

**Fig. 4:**
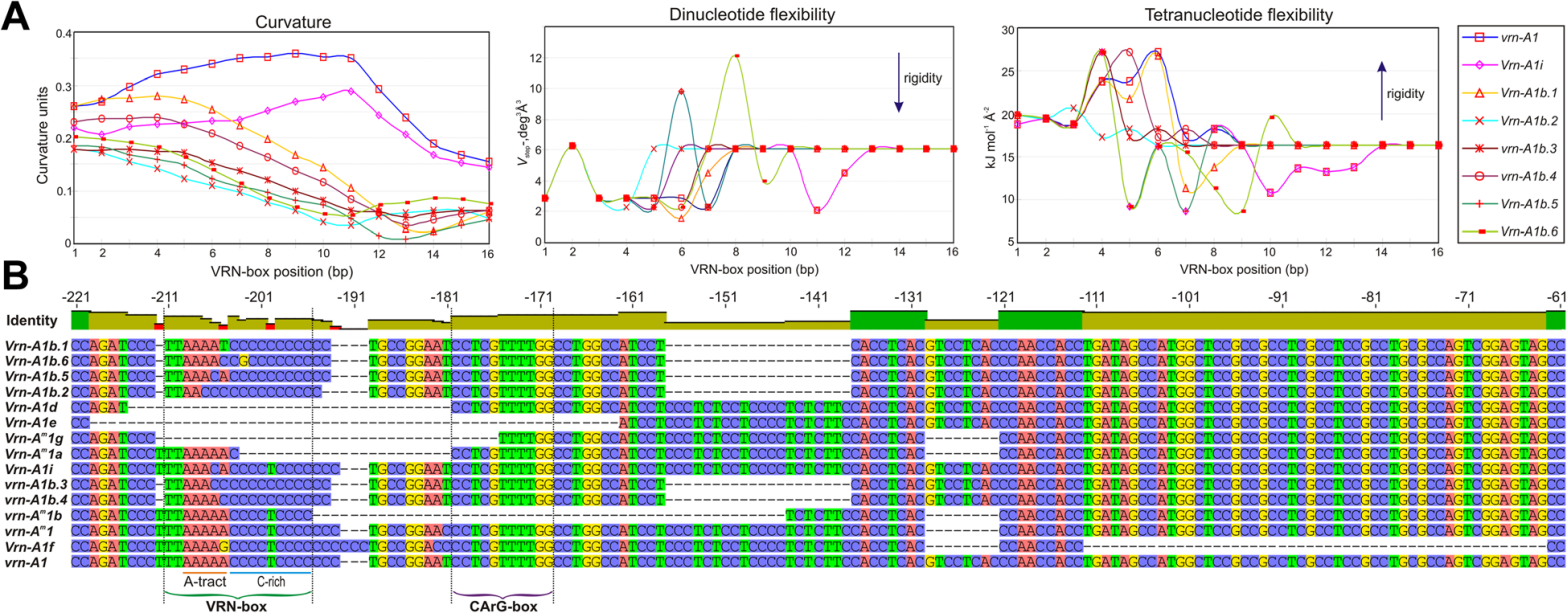
Polymorphism of the VRN-box differentiates the *VRN-A1* alleles. **a** Distribution of local curvature, dinucleotide and tetranucleotide flexibility within the VRN-box for identified here *Vrn-A1i* and variants of *Vrn-A1b*. Arrows indicate increasing rigidity of the central dinucleotide steps. **b** Multiple alignments of *VRN-A1* alleles carrying mutations within the promoter region (excluding *Vrn-A1a*); GenBank: KM047646 (*Vrn-A1b.1*), KT692944 (*Vrn-A1b.6*), KM047652 (*Vrn-A1b.5*), KM047641 (*Vrn-A1b.2*), AY616462 (*Vrn-A1d*), AY616463 (*Vrn-A1e*), DQ146422 (*Vrn-A* ^*m*^ *1g*), AY244509 (*Vrn-A* ^*m*^ *1a*), KM016790 (*Vrn-A1i*), KM047647 (*vrn-A1b.3*), KM047651 (*vrn-A1b.4*), EU875079 (*vrn-A* ^*m*^ *1b*), KM016789 (*vrn-A* ^*m*^ *1*), GQ451816 (*Vrn-A1f*), AY747600 (*vrn-A1*). The effect of deletion within *Vrn-A1f* on vernalization requirement is unclear. Positions are indicated relative to the *vrn-A1* transcription start site

Polymorphism of four promoter regions differentiates the known variants of *Vrn-A1b* and the intact *vrn-A1* allele. This includes polymorphism of the A-tract and C-rich region within the VRN-box and also a 20 bp deletion and “T” deletion downstream and upstream of the VRN-box, respectively. It was determined that deletion of the “T” upstream as well as the 20 bp deletion dowstream of the VRN-box is likely not critical for determination of the spring growth habit, because it is identified only in *Vrn-A1b* variants and absent in the other dominant *Vrn1* alleles with a mutant VRN-box, such as *Vrn-A1i*, *Vrn-A*^*m*^*1g*, *Vrn-A*^*m*^*1a*. Furthermore, *vrn-A1b.3* and *vrn-A1b.4* carrying these mutations are associated with a winter growth habit. Due to this, we assume that namely polymorphism of the VRN-box determines the decreased vernalization sensitivity of accessions which carry *Vrn-A1b*. However we cannot rule out the possible influence of mutations outside the VRN-box, which are observed in association with specific mutations within the VRN-box. The VRN-box was proposed by Pidal et al. [31] as an alternative regulatory site for vernalization. Alignments of most known *VRN-A1* alleles possessing mutations within the promoter region influencing vernalization requirement (*Vrn-A1a*, *Vrn-A1b*, *Vrn-A*^*m*^*1a*, *Vrn-A1d*, *Vrn-A1e*, *Vrn-A*^*m*^*1g*, *Vrn-A1h* and *Vrn-A1i*) have found that all contain mutations within the VRN-box such as SNPs, InDels or its total elimination (Fig. 4b). This fact emphasizes the importance of the VRN-box sequence in the transcriptional regulation of *VRN1* and in the vernalization response of wheat.

Overall, polymorphism of the VRN-box is mostly determined by mutations within A-tract and C-rich regions. Our study assumed that mutations in both of these regions influence *Vrn1* expression, detected as a difference in vernalization requirement and flowering time for accessions with a mutant VRN-box of *VRN-A1* (Table 2). The 5’-end of the VRN-box includes T-repeat and A-tract sequences. Yan et al. [19] noted that although this sequence has significant similarity to the TATA-box, it seems unlikely that this region plays an essential role in the initiation of *VRN1* transcription, because dominant alleles, which carry deletions encompassing this region have similar *VRN1* transcription levels to the intact gene. In fact, the region encompassing the A-tracts and T-repeat region (“TTAAAAA”) shows a good correlation with the TATA-box, according to the weight matrix proposed earlier by Bucher [50]. Moreover, this sequence was recognized as the TATA-box when analysing more than 1500 bp of the 5’ UTR of *VRN1* genes from B, A or D genomes using multiple independent tools for prediction of plant promoters such as TSSP [51], NSITE-PL [52] and PlantCARE [53]. In the last case, the sequence “TTTAAAAA” was identified as the core promoter element for *Zea mays* glyceraldehyde-3-phosphate dehydrogenase (*gpc2*) gene. Nevertheless, the VRN-box within the dominant *Vrn-A1d*, *Vrn-A1e* and *Vrn-A*^*m*^*1g* alleles is fully deleted. Expression of these alleles can be explained by the initiation of transcription via an alternative promoter. It is likely that transcription with either a putative alternative promoter located upstream of the VRN-box or transcription from a promoter inside the intact VRN-box is blocked by a hypothetical repressor which binds to the VRN-box sequence. Since transcription with an alternative promoter can be realized only if the VRN-box was damaged by mutations. The initiators of *VRN1* transcription, associated with insertions of genetically mobile elements within the *VRN1* promoter region and intron-1, or large deletions within the first intron, is not fully clear.

Usually it is assumed that protein affinity for DNA involves the additive contribution of nucleotides located within the target sequence. Nevertheless, the sequence-dependent features of intrinsic DNA structure and its deformability are components of so-called “indirect readout”, the principal source of non-additivity for specific protein-DNA recognition [54]. For example, TATA boxes containing 3–4 consecutive adenines have a rigid context-independent cooperative structure, which is best described by a nearest-neighbor non-additive model of protein binding [55]. According to this model the TATA-binding protein (TBP)/TATA-box system is a specific protein-DNA complex, where DNA conformation is used in extreme case and interaction is mainly through “indirect readout” [56–59]. In particular, among structural signals for TBP recognizing TATA boxes can be distinguished the helical twist angle in the middle of TATA boxes and recognition of global DNA flexibility [60]. However, in the case of A-tract polymorphism revealed in this study, the additive and non-additive components cannot be clearly separated, because the nucleotide sequence (length) of A-tract (additive component) determines flexibility and spatial structure (shape) of the DNA molecule of this sequence region (non-additive component).

Winter and spring variants of *Vrn-A1b*, and also *vrn-A1* and *Vrn-A1i*, differing by only one or several SNPs located within A-tract of VRN-box, are strongly associated with differences in vernalization requirement and flowering time. Due to this, it is assumed that polymorphism of the A-tract within the VRN-box contributes greatly to modulation of *VRN1* expression. By now it is definitively known that due to the unique structure and ability to affect intrinsic DNA curvature, the A-tracts provide a general genetic mechanism for regulation of the promoter activity by the different transcription factors and fine-tuning expression in a predictable manner, with resolution that can be even finer than that attained through altering transcription factor sites [61]. The function of such a promoter element mainly depends on its intrinsic structure, not its interaction with sequence-specific DNA-binding proteins [62]. In the present study, it was shown that polymorphism of the A-tract within the VRN-box was accompanied by a change in the curvature and flexibility of DNA molecules (Fig. 4a). Hence, it cannot be ruled out that a change in curvature and flexibility of DNA, determined by mutations of the A-tract within the VRN-box, affects the binding strength of the regulatory DNA sequence inside the VRN-box (or within flanking regions) with a hypothetical protein or protein complex that downregulates transcription of *VRN1*. For example, as was previously shown for the CArG-box, the presence of A-tracts flanking CArG-box regions can facilitate the looping of the DNA on the binding of MADS-domain transcription factors which in turn may contribute to the DNA-binding specificity of higher order complexes [63]. On the other hand, molecular dynamics simulation and experimental data revealed that the minor groove of the A-tract is a highly hydrated site, indicating that these steps have the potential to serve as a hydrogen-bonding site during protein-DNA interaction [64].

The importance of the C-rich segment within the VRN-box also cannot be excluded. The “T- > C” transition was identified only for mutant variants of *Vrn-A1b* and hence detected for “spring” (*Vrn-A1b.1*, *Vrn-A1b.2*, *Vrn-A1b.5, Vrn-A1b.6*) and “winter” (*vrn-A1b.3*, *vrn-A1b.4*) variants. However, previously it has been shown that a 20 bp deletion in the *Vrn-A*^*m*^*1a* promoter was linked to a spring growth habit [1]. This deletion includes part of C-rich region within the VRN-box (C3 instead C4TC4), which includes the site of the “T- > C” transition to 12 bp downstream. The present study revealed approximately 30 days difference between accessions with the same alleles of *VRN-B1* and *VRN-B3* genes, but differences in VRN-boxes of *VRN-A1* of only one SNP (“T- > C” transition) within the C-rich segment (*Vrn-A1b.5* and *Vrn-A1i* alleles), were found to be significant, with a more later flowering phenotype characterized for accessions with an intact C-rich region (*Vrn-A1i* allele). Furthermore, the dominant allele *Vrn-A1b.6* differs from the recessive *vrn-A1b.4* by only one SNP within the C-rich segment. Finally, almost all dominant alleles of *Vrn1* with a mutant VRN-box carry mutations within the C-rich segment (Fig. 4b). Only *Vrn-A1i* has an intact C-rich, region, and this was associated with a later flowering time. These data emphasize the importance of the C-rich sequence within the VRN-box in repression of *VRN-A1* transcription. It is known that guanine- and cytosine-rich regions of DNA are able to form a non-canonical nucleic acid substructure conformations comprising four-stranded DNA secondary structures, namely the G-quadruplex and i-motif. These structures are involved in the control of gene expression through regulation of transcription activity [65, 66] or post-transcriptional regulation [67]. Hence, it can be hypothesised that the C-rich region of the VRN-box regulates *Vrn1* transcription through formation of quadruplex structures which are destabilized by a “T- > C” transition (for variants of *Vrn-A1b*) or cannot be formed due to almost full deletion of the nucleotide sequence (for *Vrn-A*^*m*^*1a*) accompanying transcription activation of *VRN1*. For example, mutational destabilization of the C-MYC (human oncogene) promoter G-quadruplex leads to greater transcriptional activity due to the destabilization of a DNA-protein complex, where the protein is a transcriptional repressor [68]. Furthermore, there is an opinion that G-tracts have transcriptional properties virtually identical to those of A-tracts [62]. Interestingly, according to biophysical stability studies the formation of G-quadruplex and i-motif conformations leads to destabilization of the proximal duplex regions [69] that can be the basis for interactions between the C-rich segment and A-tract within the VRN-box. Although with less similarity than a quadruplex motif, C-rich region of VRN-box (as well as the 20 bp deletion downstream of the VRN-box for *Vrn-A1b* alleles) is also associated with a core promoter element, the so-called Y-Patch, a CT-rich region often found in plant TATA box promoters [70]. For example, around 50 % of rice core promoters contain one or more Y-Patches [71].

## Conclusions

The *VRN-A1* and *VRN-B1* genes were analysed in hexaploid and tetraploid wheat species. During investigation the seven novel variants of *VRN-A1* and three promoter sequence variants of *VRN-B1* were identified. For detection of these variants during PAGE of PCR fragments the electrophoretic conditions were optimized for low and room temperature. Novel allelic variants of *VRN-A1* identified herein are more frequent compared to previously known *VRN-A1* alleles. It is assumed that polymorphism of the A-tract within the VRN-box of the *VRN-A1* gene is associated with modulation of the vernalization requirement and flowering time of wheat. Furthermore, it is apparent that the C-rich segment and the adjacent A-tract, both contribute to the functionality of the VRN-box. *VRN-B1.s* was associated with early flowering of the spring wheat accessions. In summary, the results of our study not only enrich the genetic source material for wheat breeding with new agronomically valuable alleles, but also provide a set of natural mutants for fundamental investigations essential to achieving a better understanding of the mechanisms of the regulation of *VRN1* expression in wheat.

## Methods

### Plant material

A total of 178 accessions representing 5 hexaploid and 6 tetraploid wheat species from 57 countries and different eco-geographic areas were investigated (Additional file 2: Table S1, Additional file 3: Table S2). Hexaploid species (genome BBA^u^A^u^DD) included were *T. spelta* L., *T. macha* Dekap, *T. vavilovii* Jakubz, *T. compactum* Host, *T. sphaerococcum* Percival. Tetraploid species (genome BBA^u^A^u^) consisted of *T. durum* Desf., *T. turgidum* L., *T. polonicum* L., *T. carthlicum* Nevski, *T. dicoccum* Schrank and *T. dicoccoides* Körn. Germplasm was obtained from the National Plant Germplasm System (NPGS, USA) and National Center of Plant Genetic Resources (Ukraine).

The experimental plants were grown in a photoperiod-controlled glasshouse under 16 h of natural light (long day conditions). Days to flowering were registered for each accession. The plants that failed to reach stem elongation by the end of the experiment (120 days) were considered to have a winter growth habit.

### DNA extraction and PCR amplification

Total DNA from 4-day-old wheat seedlings was extracted following a modified CTAB-method [72]. PCR reactions consisted of: DNA (~40 ng), 20 mM Tris–HCl (pH 8.8), 10 mM (NH_2_)_2_SO_4_, 2.6 mM MgCl_2_, 1 mM KCl, 0.1 % Triton X-100, 250 μM dNTPs, 1.5 % DMSO, 3 ng/uL each primer, 0.05 U/uL Taq-polymerase. PCR was performed using the following program: initial denaturation at 94 °C (2 min); 30 cycles of amplification: 94 °C (10 s), annealing (10 s), 74 °C (50 s or 1.5 min for amplification of the *VRN-B1* intron-1) per cycle, followed by 3 cycles of: annealing (10 s), 74 °C (40 s) and a final elongation step of 72 °C for 3 min. Further details of all primers, including annealing temperatures, are listed in Table 1.

### Electrophoresis in polyacrylamide and agarose gels

Amplification products of the *VRN-A1* and *VRN-B1* first intron, *VRN-B3* alleles and variants of *Vrn-A1a* were separated on 6.6 % nondenaturing polyacrylamide (PAA) gels (mono/bis-acrylamide ratio 82:1) in 1.38 TBE buffer (123 mM ionic strength), at room temperature, under 6 V/cm until the bands had migrated 50–70 % of the length of the gel. To enable detection of the allelic variants of *VRN-A1* and *VRN-B1* by PCR fragments of the *VRN1* promoter region the conditions of polyacrylamide gel electrophoresis (PAGE) were optimized for low and room temperature to obtain the minimal loss of resolution under these conditions (Additional file 4). Thus electrophoresis in 6.6 % polyacrylamide gels at mono/bis-acrylamide ratio 82:1, under low electric field (2.5 V/cm) and high ionic strength (123 mM, x1.38 TBE in gel) was carried out at room temperature, while the best results for a short duration were obtained when electrophoresis was run at low temperature (10–20 °C) with high electric field strength (5–10 V/cm). Visualization of PCR fragments in PAA gels was conducted using a modified silver staining protocol [73]. Agarose gel electrophoresis was performed using 1.5 % agarose in x1 TBE buffer. PCR products were stained with ethidium bromide and viewed under UV light.

### Cloning and sequencing of PCR fragments

PCR amplicons were separated on 1.5 % agarose gels, gel-extracted using a GeneJET Gel Extraction Kit (Thermo Scientific), and DNA fragments ligated into pGEM-T (Promega), according to manufacturer’s instructions. Plasmid DNA was transformed into JM109 *Escherichia coli* competent cells (L2001, Promega). Transformed cells containing the plasmid carrying an insertion of foreign DNA fragment were detected using white-blue selection on growth medium containing ampicillin, X-Gal, and IPTG. Positive colonies were tested for the presence of cloned PCR products by PCR with universal pUC primers (forward and reverse M13 primers), followed by separation and visualisation of PCR products on agarose gels. Plasmid DNA was extracted using GeneJET Plasmid Miniprep Kit (Thermo Scientific), and sequencing PCRs performed using an ABI3700 Bioanalyser (Applied Biosystems), following the manufacturer’s protocol. Fluorescently labelled extension products were precipitated and resuspended in HiDi Formamide (Applied Biosystems).

### Nomenclature

The different sequence variants of the *Vrn-A1b* allele were designated according to a numerical index, i.e. “*b.1*”, “*b.2*”, etc. A similar convention was employed for variants of *Vrn-A1a*, which were be identified by the specific combination of PCR fragments of certain lengths. Sequence variants of the *VRN-B1* promoter region were designated by the literal index “f”, “m” and “s”, indicating respectively a “fast”, “medium” or “slow” relative migration rate of the associated PCR fragments in PAA gels. These sequence variants combined with the different *VRN-B1* alleles (*vrn-B1*, *Vrn-B1a* and *Vrn-B1c*) with the exception of *Vrn-B1(ins)*. The partial nucleotide sequences of *VRN-A1* and *VRN-B1* (promoter region) reported in this paper were deposited in GenBank under the accessions: KR782255 (*Vrn-A1a.2*), KM016789 (*vrn-A*^*m*^*1*), KM016790-KM016792 (*Vrn-A1i*), KT361213 (*Vrn-A1e*), KM047646 (*Vrn-A1b.1*), KM047641-KM047645 (*Vrn-A1b.2*), KM047647-KM047650 (*vrn-A1b.3*), KM047651 (*vrn-A1b.4*), KM047652 (*Vrn-A1b.5*), KT692944-KT692945 (*Vrn-A1b.6*), KR782252 (*VRN-B1.s*), KT361212 (*VRN-B1.m*), KR782253-KR782254 (*VRN-B1.f*).

### Data analyses

For estimation of intrinsic curvature and local bend angles, three dimensional DNA models describing the trajectory of the contour path, central axis and phosphates were generated. The calculation of reconstructed DNA trajectory and the evaluation of DNA curvature was performed according to previously published methods (in Methods section of [42]). Structures were visualized by the PyMOL Molecular Graphics System, Version 1.7.2 Schrödinger, LLC. Distribution of flexibility of DNA molecules was evaluated based on the relevant parameters for di- and tetranucleotide steps published earlier [74, 75]. Multiple sequence alignments were generated using Clustal W [76]. The *T. aestivum* ESTs database (UniGene Build #63) containing 1551792 sequences in 178464 clusters was downloaded from NCBI (ftp://ftp.ncbi.nih.gov/repository/UniGene/Triticum_aestivum/Ta.seq.all.gz). A sequence similarity search was performed using BLAST+ software [77]. RNA secondary structure was predicted using the RNAstructure web service [78].

## Additional files

Additional file 1: Figure S1. “Distribution of curvature and bending angles for PCR fragments of *vrn-A1* and *Vrn-A1i*”. (DOC 1683 kb) Additional file 2: Table S1. “The *VRN-A1* and *VRN-B1* alleles of hexaploid wheat accessions”. (DOC 118 kb) Additional file 3: Table S2. “The *VRN1* alleles identified for tetraploid wheat accessions”. (DOC 141 kb) Additional file 4:
**“Effect of electrophoretic conditions on discrimination of** ***VRN-A1*** **and** ***VRN-B1*** **alleles”.** “Optimization of PAGE for detection of the *VRN-A1* and *VRN-B1* alleles”. (DOC 1476 kb)
