# Supplementary material for: Novel alleles of the VERNALIZATION1 genes in wheat are associated with modulation of DNA curvature and flexibility in the promoter region
Source: BMC Plant Biol. 2016 Jan 27;16(Suppl 1):9. doi: 10.1186/s12870-015-0691-2 (PMC4895274; doi:10.1186/s12870-015-0691-2)

**Novel alleles of the *VERNALIZATION1* genes in wheat are associated with modulation of DNA curvature and flexibility in the promoter region**

**Additional file 1**

**Figure S1. Distribution of curvature and bending angles for PCR fragments of *vrn-A1* and *Vrn-A1i*.**


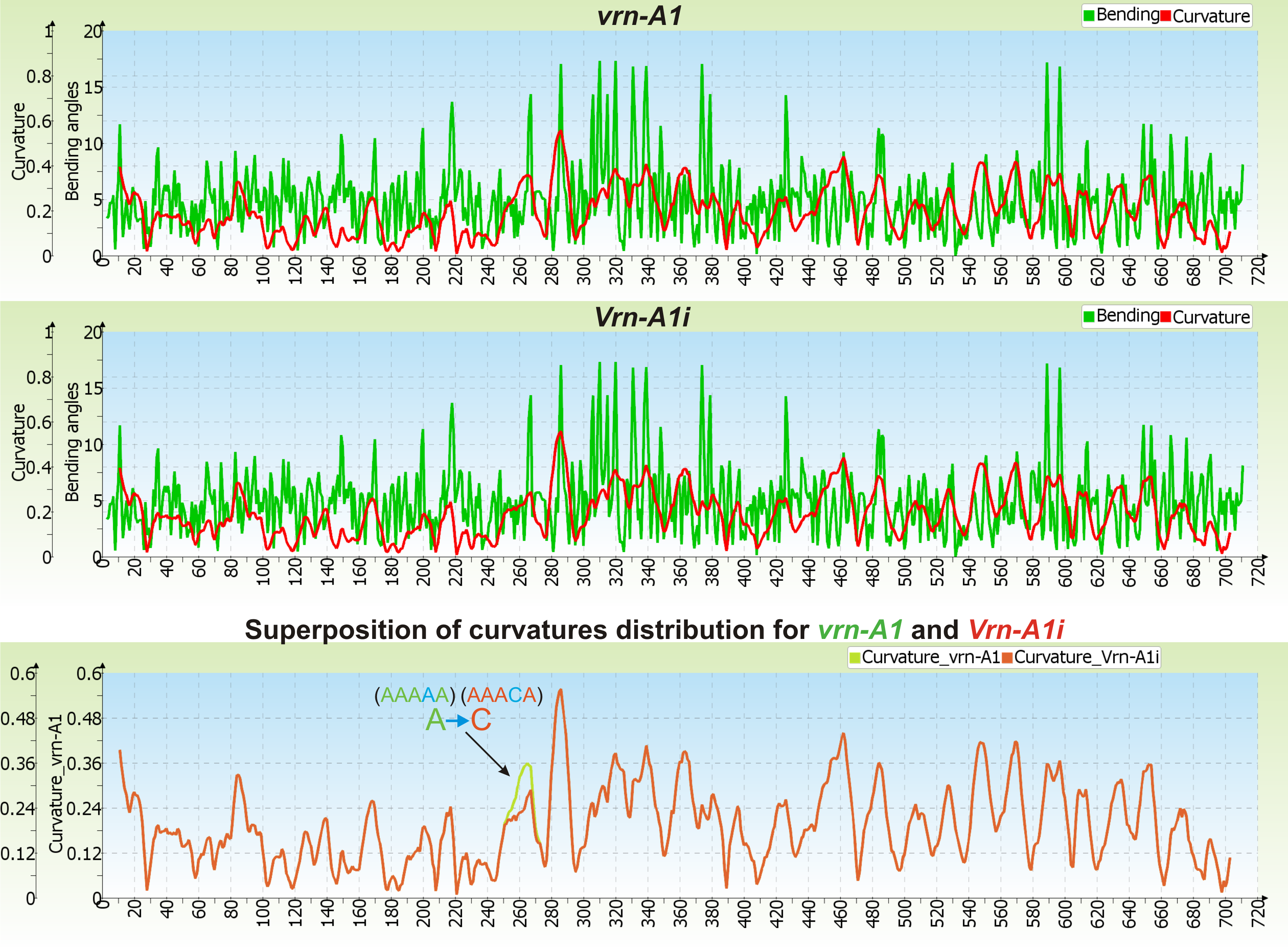

Supplement: Additional file 1: Figure S1. — “Distribution of curvature and bending angles for PCR fragments of vrn-A1 and Vrn-A1i”. (DOC 1683 kb) [file 12870_2015_691_MOESM1_ESM.doc]
