# Supplementary material for: Novel alleles of the VERNALIZATION1 genes in wheat are associated with modulation of DNA curvature and flexibility in the promoter region
Source: BMC Plant Biol. 2016 Jan 27;16(Suppl 1):9. doi: 10.1186/s12870-015-0691-2 (PMC4895274; doi:10.1186/s12870-015-0691-2)
Supplement: Additional file 2: Table S1. — “The VRN-A1 and VRN-B1 alleles of hexaploid wheat accessions”. (DOC 118 kb) [file 12870_2015_691_MOESM2_ESM.doc]

**Novel alleles of the *VERNALIZATION1* genes in wheat are associated with modulation of DNA curvature and flexibility in the promoter region**

**Additional file 2**

**Allelic variants at the *VRN1* genes for hexaploid wheat**

**Notes for Tables S1**

*Vrn-A1a.1* and *Vrn-A1a.3* previously identified by Yan et al. 2004 and known as *Vrn-A1a*;

*Vrn-A1a.2* identified in this study;

*Vrn-A1i* identified in this study;

*Vrn-A1b.1* corresponds to the original *Vrn-A1b* allele (GenBank: AY616461);

*Vrn-A1b.2-b.6* sequence variants of *Vrn-A1b* identified in this study;

*vrn-A1b.3* and *vrn-A1b.4* associated with a winter growth habit;

For *VRN-B1* in brackets the promoter sequence variant is indicated (*VRN-B1.f* – intact promoter (corresponds to *vrn-B1* allele); *VRN-B1.s –* promoter contains 7, 3 and 2 bp deletions; *VRN-B1.m* – promoter contains 3 and 2 bp deletions).

**Table S1. The *VRN-A1* and *VRN-B1* alleles of hexaploid wheat accessions. The identification of these accessions at the *VRN-D1* allelic variants available on supplementary material our previous study [1].**

| **Species** | **Accession**  **name** | **Accession**  **ID** | **Country** | ***VRN-A1***  **alleles** | ***VRN-B1***  **alleles** |
| --- | --- | --- | --- | --- | --- |
| *Triticum sphaerococcum*  Percival. | 23824 | PI 115818 | India | *vrn-A1* | *vrn-B1 (VRN-B1.f)* |
|  | 52 | CItr 8610 | China | *vrn-A1* | *vrn-B1 (VRN-B1.f)* |
|  | S-49 | PI 182118 | Pakistan | *vrn-A1* | *vrn-B1 (VRN-B1.f)* |
|  | 2531 | PI 190982 | Belgium | *vrn-A1* | *vrn-B1 (VRN-B1.f)* |
|  | Sahari M.3 | PI 191301 | Portugal | *vrn-A1* | *vrn-B1 (VRN-B1.f)* |
|  | I-1-3572 | PI 272580 | Hungary | *vrn-A1* | *Vrn-B1c (VRN-B1.f)* |
|  | Acarp | PI 277142 | India | *vrn-A1* | *vrn-B1 (VRN-B1.f)* |
|  | 971 | PI 278650 | UK | *vrn-A1* | *vrn-B1 (VRN-B1.f)* |
|  | 219 | PI 70711 | Iraq | *vrn-A1* | *vrn-B1 (VRN-B1.f)* |
|  | I12 | PI 83402 | China | *vrn-A1* | *vrn-B1 (VRN-B1.f)* |
|  | - | PI 324492 | India | *vrn-A1* | *vrn-B1 (VRN-B1.f)* |
|  | Type No. 4 | PI 40941 | Pakistan | *vrn-A1* | *vrn-B1 (VRN-B1.f)* |
|  | 125 | PI 42013 | India | *vrn-A1* | *vrn-B1 (VRN-B1.f)* |
|  | S 2130 | PI 277141 | Germany | *vrn-A1* | *vrn-B1 (VRN-B1.f)* |
|  | CI 12212 | PI 168685 | USA | *vrn-A1* | *vrn-B1 (VRN-B1.f)* |
|  | CI 17737 | CItr 17737 | USA | *vrn-A1* | *vrn-B1 (VRN-B1.f)* |
|  | Rubriglabrum | UA0300243 | Pakistan | *vrn-A1* | *vrn-B1 (VRN-B1.f)* |
|  | Tumidum | UA0300244 | India | *vrn-A1* | *vrn-B1 (VRN-B1.f)* |
| *Triticum compactum* Host. | Gluclub | PI 114638 | Australia | *Vrn-A1b.6* | *Vrn-B1a (VRN-B1.f)* |
|  | PS 1785-847 | PI 186391 | China | *vrn-A1* | *vrn-B1 (VRN-B1.f)* |
|  | Velino | PI 157920 | Italy | *vrn-A1* | *vrn-B1 (VRN-B1.f)* |
|  | Ostka Skomoroska | PI 129523 | Poland | *vrn-A1* | *vrn-B1 (VRN-B1.f)* |
|  | Spitskop | PI 159101 | South Africa | *vrn-A1* | *Vrn-B1a (VRN-B1.f)* |
|  | Kanak | PI 164160 | India | *Vrn-A1b.6* | *Vrn-B1a (VRN-B1.f)* |
|  | H 23 H 13385 | PI 191542 | Portugal | *Vrn-A1b.6* | *vrn-B1 (VRN-B1.s)* |
|  | 1413-1 | PI 211701 | Turkey | *vrn-A1* | *vrn-B1 (VRN-B1.f)* |
|  | Aleppo 23 | PI 278541 | Syria | *vrn-A1* | *Vrn-B1a (VRN-B1.f)* |
|  | Kozha Bidai | PI 262666 | Kazakhstan | *vrn-A1, Vrn-A1b.6* | *Vrn-B1a (VRN-B1.f)* |
|  | Premier | PI 278581 | UK | *vrn-A1* | *vrn-B1 (VRN-B1.s)* |
|  | Bivona | PI 294567 | USA | *Vrn-A1a.1* | *vrn-B1 (VRN-B1.s)* |
|  | Herrison Sans Barbe | PI 294892 | Bulgaria | *Vrn-A1a.1* | *vrn-B1 (VRN-B1.s)* |
|  | Rouge de la Gruyere | PI 352298 | Switzerland | *Vrn-A1a.2* | *Vrn-B1a (VRN-B1.f)* |
|  | Tiroler Fruhe Binkel | PI 352302 | Austria | *Vrn-A1a.2* | *Vrn-B1a (VRN-B1.f)* |
|  | Ble DAvril | PI 352306 | France | *vrn-A1* | *vrn-B1 (VRN-B1.f)* |
|  | DN-2263 | PI 361807 | Denmark | *vrn-A1* | *vrn-B1 (VRN-B1.f )* |
|  | W44A | PI 410652 | Pakistan | *vrn-A1* | *Vrn-B1a (VRN-B1.f )* |
|  | 134 | PI 436279 | Chile | *Vrn-A1b.6* | *vrn-B1 (VRN-B1.s)* |
|  | Termok | PI 41023 | Kyrgyzstan | *vrn-A1, Vrn-A1b.6* | *Vrn-B1a (VRN-B1.f)* |
|  | Erinaccum | UA0300240 | Armenia | *Vrn-A1a.2* | *Vrn-B1a (VRN-B1.f)* |
|  | Grizeoicterinum | UA0300245 | USA | *Vrn-A1a.1* | *Vrn-B1a (VRN-B1.f)* |
| *Triticum spelta* L. | Duhamelianum, Frankenkorn | UA0300103 | Australia | *vrn-A1* | *vrn-B1* (*VRN-B1.f)* |
|  | Duhamelianum, NSS 1/02 | UA0300259 | Serbia | *vrn-A1* | *vrn-B1* (*VRN-B1.f)* |
|  | Album, NSS 1/01 | UA0300246 | Serbia | *vrn-A1* | *Vrn-B1c* (*VRN-B1.f)* |
|  | Caeruleum, Tridentina | UA0300218 | Italy | *Vrn-A1b.2* | *vrn-B1* (*VRN-B1.s)* |
|  | Album | UA0300304 | Australia | *Vrn-A1b.2* | *Vrn-B1c* (*VRN-B1.f)* |
|  | Bearded Spelt | PI 168680 | USA | *vrn-A1b.3* | *Vrn-B1c* (*VRN-B1.f)* |
|  | Lignee 10 | PI 190960 | Belgium | *vrn-A1* | *vrn-B1* (*VRN-B1.f)* |
|  | 2670 | PI 190962 | Italy | *Vrn-A1b.2* | *Vrn-B1c* (*VRN-B1.f )* |
|  | Spelta Hohenheim | PI 190963 | Portugal | *vrn-A1* | *vrn-B1* (*VRN-B1.f )* |
|  | Album | PI 221419 | Serbia | *Vrn-A1b.2* | *Vrn-B1c* (*VRN-B1.f )* |
|  | 85 | PI 225271 | Iran | *vrn-A1* | *vrn-B1* (*VRN-B1.f)* |
|  | I-1-599 | PI 272573 | Hungary | *Vrn-A1b.2* | *Vrn-B1c* (*VRN-B1.f)* |
|  | Stauderers Markus | PI 286048 | Germany | *vrn-A1* | *vrn-B1* (*VRN-B1.f)* |
|  | Gruzia | PI 295056 | Bulgaria | *vrn-A1* | *vrn-B1* (*VRN-B1.f)* |
|  | 2943 | PI 306550 | Romania | *vrn-A1b.3* | *Vrn-B1c* (*VRN-B1.f)* |
|  | - | PI 323438 | Austria | *vrn-A1b.3* | *Vrn-B1c* (*VRN-B1.f )* |
|  | Coeruleum | PI 330558 | UK | *Vrn-A1b.2* | *vrn-B1* (*VRN-B1.s)* |
|  | Altgold | PI 347850 | Switzerland | *vrn-A1* | *vrn-B1* (*VRN-B1.f)* |
|  | 69Z6.602 | PI 348428 | Spain | *Vrn-A1b.2* | *vrn-B1* (*VRN-B1.s)* |
|  | 26867-302Y-300M-OY | PI 520066 | Mexico | *Vrn-A1b.6* | *vrn-B1* (*VRN-B1.f)* |
|  | WIR 52470 | PI 572914 | Tajikistan | *vrn-A1* | *vrn-B1* (*Vrn-B1f )* |
|  | Altgold | PI 355629 | Switzerland | *vrn-A1* | *vrn-B1* (*VRN-B1.f )* |
|  | Babenhauser Rotvesen | PI 355633 | Germany | *vrn-A1* | *vrn-B1* (*VRN-B1.f )* |
|  | 69Z6.884 | PI 348700 | Spain | *Vrn-A1b.2* | *vrn-B1* (*VRN-B1.s)* |
| *Triticum*  *macha* Dekapr. | I-1-2710 | PI 272554 | Hungary | *vrn-A1* | *vrn-B1* (*VRN-B1.f)* |
|  | Letshchumicum | PI 352466 | FSU | *vrn-A1* | *vrn-B1* (*VRN-B1.f)* |
|  | 69Z5.190 | PI 355511 | Russia | *vrn-A1* | *vrn-B1* (*VRN-B1.f)* |
|  | 69Z5.193 | PI 355514 | Switzerland | *vrn-A1* | *vrn-B1* (*VRN-B1.f)* |
|  | DN-2378 | PI 361862 | Denmark | *vrn-A1* | *vrn-B1* (*VRN-B1.f)* |
|  | G532 | PI 428146 | Sweden | *vrn-A1* | *vrn-B1* (*VRN-B1.f)* |
|  | G866 | PI 428178 | Italy | *Vrn-A1b.2* | *Vrn-B1c* (*VRN-B1.f)* |
|  | G1569 | PI 428179 | Iran | *vrn-A1* | *vrn-B1* (*VRN-B1.f)* |
|  | H86-708 | PI 542466 | USA | *vrn-A1* | *vrn-B1* (*VRN-B1.f)* |
|  | WIR 29576 | PI 572905 | Georgia | *vrn-A1* | *vrn-B1* (*VRN-B1.f)* |
| *Triticum*  *vavilovii* Jakubz. | WIR 29533 | PI 326319 | Armenia | *vrn-A1* | *vrn-B1* (*VRN-B1.f )* |
|  | - | PI 428342 | Sweden | *vrn-A1b.3* | *vrn-B1* (*VRN-B1.f)* |
|  | - | PI 428343 | Sweden | *vrn-A1* | *vrn-B1* (*VRN-B1.f )* |

**References**

1. Muterko A, Balashova I, Cockram J, Kalendar R, Sivolap Y (2015) The new wheat vernalization response allele *Vrn-D1s* is caused by DNA transposon insertion in the first intron. Plant Mol Biol Rep 33(2):294-303.
