# Supplementary material for: Novel alleles of the VERNALIZATION1 genes in wheat are associated with modulation of DNA curvature and flexibility in the promoter region
Source: BMC Plant Biol. 2016 Jan 27;16(Suppl 1):9. doi: 10.1186/s12870-015-0691-2 (PMC4895274; doi:10.1186/s12870-015-0691-2)
Supplement: Additional file 3: Table S2. — “The VRN1 alleles identified for tetraploid wheat accessions”. (DOC 141 kb) [file 12870_2015_691_MOESM3_ESM.doc]

**Novel alleles of the *VERNALIZATION1* genes in wheat are associated with modulation of DNA curvature and flexibility in the promoter region**

**Additional file 3**

**Allelic variants at the *VRN1* genes for tetraploid wheat**

**Notes for Tables S2**

*Vrn-A1a.1* and *Vrn-A1a.3* previously identified by Yan et al. 2004 and known as *Vrn-A1a*;

*Vrn-A1a.2* identified in this study;

*Vrn-A1i* identified in this study;

*Vrn-A1b.1* corresponds to the original *Vrn-A1b* allele (GenBank: AY616461);

*Vrn-A1b.2-b.6* sequence variants of *Vrn-A1b* identified in this study;

*vrn-A1b.3* and *vrn-A1b.4* associated with a winter growth habit;

For *VRN-B1* in brackets the promoter sequence variant is indicated (*VRN-B1.f* – intact promoter (corresponds to *vrn-B1* allele); *VRN-B1.s –* promoter contains 7, 3 and 2 bp deletions; *VRN-B1.m* – promoter contains 3 and 2 bp deletions).

**Table S2. The *VRN1* alleles identified for tetraploid wheat accessions.**

| **Species** | **Accession**  **name** | **Accession**  **ID** | | **Country** | ***VRN-A1***  **alleles** | ***VRN-B1***  **alleles** |
| --- | --- | --- | --- | --- | --- | --- |
| *Triticum*  *carthlicum* Nevski. | 11891 | PI 115817 | | Georgia | *vrn-A1* | *Vrn-B1ins* |
|  | - | PI 168672 | | China | *Vrn-A1c* | *vrn-B1 (VRN-B1.f)* |
|  | 3772 | PI 190949 | | Portugal | *vrn-A1* | *Vrn-B1ins* |
|  | I-1-2715 | PI 272521 | | Hungary | *vrn-A1* | *Vrn-B1ins* |
|  | CPI 2679 | PI 283887 | | Iran | *vrn-A1* | *Vrn-B1ins* |
|  | - | PI 286070 | | Poland | *vrn-A1* | *Vrn-B1ins* |
|  | WIR 32510 | PI 341800 | | Russia | *vrn-A1* | *Vrn-B1ins* |
|  | WIR 13810 | PI 349040 | | Armenia | *Vrn-A1e* | *Vrn-B1ins* |
|  | T-1513 | PI 352279 | | FSU | *vrn-A1* | *Vrn-B1ins* |
|  | H83-1579 | PI 532502 | | USA | *Vrn-A1e* | *Vrn-B1ins* |
|  | H83-1538 | PI 532505 | | Canada | *vrn-A1* | *Vrn-B1ins* |
|  | H84-558-3 | PI 532512 | | UK | *vrn-A1* | *Vrn-B1ins* |
| *Triticum dicoccoides* Körn. | - | PI 233288 | | Israel | *Vrn-A1b.2* | *vrn-B1, Vrn-B1c (VRN-B1.f, VRN-B1.s)* |
|  | DS-9 | PI 256029 | | Spain | *Vrn-A1b.2* | *vrn-B1 (VRN-B1.s)* |
|  | 84 | PI 266841 | | UK | *Vrn-A1b.2* | *vrn-B1 (VRN-B1.s)* |
|  | I-1-2708 | PI 272582 | | Hungary | *Vrn-A1b.2* | *Vrn-B1c (VRN-B1.f)* |
|  | Vavilovii | PI 352322 | | Lebanon | *Vrn-A1b.2* | *Vrn-B1c (VRN-B1.m)* |
|  | Kotschyi | PI 352323 | | Asia Minor | *vrn-A1* | *vrn-B1 (VRN-B1.s)* |
|  | Spontaneo villosum | PI 352325 | | Switzerland | *vrn-A1* | *vrn-B1 (VRN-B1.f)* |
|  | Spontaneo villosum | PI 352326 | | Germany | *Vrn-A1b.2* | *Vrn-B1c (VRN-B1.f)* |
|  | Psendogord | PI 362036 | | Romania | *Vrn-A1b.2* | *Vrn-B1c (VRN-B1.f)* |
|  | G2041 | PI 428018 | | Turkey | *vrn-A1* | *vrn-B1 (VRN-B1.f)* |
|  | A-54 | PI 466941 | | Syria | *vrn-A1b.4* | *vrn-B1 (VRN-B1.m)* |
|  | Namuricum | UA0300256 | | Jordan | *vrn-A1* | *vrn-B1 (VRN-B1.f)* |
| *Triticum*  *dicoccum* Schrank. | 2323A | PI 190920 | | Portugal | *Vrn-A1b.2* | *vrn-B1 (VRN-B1.f)* |
|  | 2467 | PI 190921 | | Belgium | *Vrn-A1b.2* | *vrn-B1 (VRN-B1.s)* |
|  | Escanda de Malvedo | PI 191091 | | Spain | *Vrn-A1a.3* | *vrn-B1 (VRN-B1.f)* |
|  | 119 | PI 276015 | | Spain | *Vrn-A1a.3* | *vrn-B1 (VRN-B1.f)* |
|  | Serbicum | UA0300183 | | Russian | *Vrn-A1a.1* | *Vrn-B1c (VRN-B1.f)* |
|  | - | UA0300013 | | Syrian | *Vrn-A1a.1* | *Vrn-B1c (VRN-B1.f)* |
|  | - | UA0300003 | | Germany | *Vrn-A1b.2* | *vrn-B1 (VRN-B1.s)* |
|  | - | UA0300082 | | Germany | *Vrn-A1b.2* | *vrn-B1 (VRN-B1.s)* |
|  | Aeruginosum | UA0300027 | | Russian | *Vrn-A1e* | *vrn-B1 (VRN-B1.f)* |
|  | Pychurum | UA0300083 | | Germany | *Vrn-A1b.2* | *vrn-B1 (VRN-B1.s)* |
|  | Unimiegei | UA0300212 | | Morocco | *Vrn-A1b.5* | *vrn-B1 (VRN-B1.s)* |
|  | Atratum | UA0300214 | | USA | *vrn-A1b.3* | *vrn-B1 (VRN-B1.f)* |
| *Triticum*  *durum* Desf. | 452 | PI 94732 | | Italy | *Vrn-A1b.1* | *vrn-B1 (VRN-B1.s)* |
|  | 454 | PI 94733 | | Ethiopia | *Vrn-A1c* | *vrn-B1 (VRN-B1.s)* |
|  | 409 | PI 94710 | | FSU | *Vrn-A1c* | *Vrn-B1a (VRN-B1.f)* |
|  | 434 | PI 94722 | | Asia Minor | *Vrn-A1c* | *vrn-B1 (VRN-B1.f)* |
|  | 429 | PI 94721 | | Portugal | *Vrn-A1c* | *vrn-B1 (VRN-B1.f)* |
|  | 427 | PI 94720 | | Spain | *Vrn-A1c* | *vrn-B1 (VRN-B1.f)* |
|  | Melanopus 69 | PI 94729 | | Russia | *Vrn-A1c* | *vrn-B1 (VRN-B1.f)* |
|  | 424 | PI 94578 | | Egypt | *Vrn-A1c* | *vrn-B1 (VRN-B1.f)* |
|  | 394 | PI 94705 | | Palestine | *Vrn-A1c* | *vrn-B1 (VRN-B1.s)* |
|  | Khandwa | PI 8898 | | India | *Vrn-A1c* | *vrn-B1 (VRN-B1.f)* |
|  | Marching No. 8 | PI 81792 | | Japan | *Vrn-A1c* | *vrn-B1 (VRN-B1.f)* |
|  | N-85 | PI 79900 | | China | *Vrn-A1c* | *vrn-B1 (VRN-B1.f)* |
|  | - | PI 88737 | | Greece | *Vrn-A1b.6* | *vrn-B1 (VRN-B1.s)* |
|  | 2912 | PI 74830 | | China | *Vrn-A1i* | *vrn-B1 (VRN-B1.s)* |
|  | Abd-el-Kader | PI 7653 | | Tunisia | *Vrn-A1c* | *vrn-B1 (VRN-B1.f)* |
|  | OR2010539 | PI 655432 | | USA | *vrn-A1b.3* | *vrn-B1 (VRN-B1.f)* |
|  | Mongolian | CItr 10024 | | USA | *Vrn-A1b.6* | *vrn-B1 (VRN-B1.f)* |
|  | Vallega Zittelli 486 | CItr 15100 | | Italy | *Vrn-A1c* | *vrn-B1 (VRN-B1.f)* |
|  | Wascana | CItr 15280 | | Canada | *Vrn-A1c* | *vrn-B1 (VRN-B1.f)* |
|  | Royal de Almena | CItr 15278 | | Argentina | *Vrn-A1c* | *vrn-B1 (VRN-B1.s)* |
|  | Hazera 1203 | CItr 15274 | | Israel | *Vrn-A1b.1* | *vrn-B1 (VRN-B1.s)* |
| *Triticum*  *polonicum* L. | Martinari | PI 134945 | | Portugal | *Vrn-A1b.6* | *vrn-B1 (VRN-B1.f)* |
|  | Mika | PI 167622 | | Turkey | *Vrn-A1b.6* | *vrn-B1 (VRN-B1.f)* |
|  | Polonicum | PI 185309 | | Argentina | *Vrn-A1b.6* | *vrn-B1 (VRN-B1.f)* |
|  | Sin El-Pheel | PI 208911 | | Iraq | *Vrn-A1b.6* | *vrn-B1 (VRN-B1.f)* |
|  | 7959 | PI 210845 | | Iran | *Vrn-A1c* | *vrn-B1 (VRN-B1.s)* |
|  | Tafeelih Riti | PI 223171 | | Jordan | *Vrn-A1b.6* | *vrn-B1 (VRN-B1.f)* |
|  | 3002 | PI 245663 | | Afghanistan | *Vrn-A1c* | *vrn-B1 (VRN-B1.s)* |
|  | 2719 | PI 254214 | | India | *Vrn-A1b.6* | *vrn-B1 (VRN-B1.f)* |
|  | 88 | PI 266846 | | UK | *Vrn-A1c* | *vrn-B1 (VRN-B1.s)* |
|  | I-1-3496 | PI 272564 | | Hungary | *Vrn-A1c* | *vrn-B1 (VRN-B1.s)* |
|  | Assyrian Rye | PI 29447 | | Ukraine | *Vrn-A1c* | *vrn-B1 (VRN-B1.s)* |
|  | 2939 | PI 306548 | | Romania | *Vrn-A1c* | *vrn-B1 (VRN-B1.s)* |
|  | WIR 42758 | PI 349052 | | Azerbaijan | *Vrn-A1c* | *vrn-B1 (VRN-B1.s)* |
|  | T-742 | PI 352487 | | Germany | *Vrn-A1c* | *vrn-B1 (VRN-B1.s)* |
|  | Mirabella | PI 352488 | | Italy | *Vrn-A1c* | *vrn-B1 (VRN-B1.s)* |
|  | T-1515 | PI 352489 | | Cyprus | *Vrn-A1c* | *vrn-B1 (VRN-B1.s)* |
|  | 42-1 | PI 384265 | | Ethiopia | *Vrn-A1c* | *vrn-B1 (VRN-B1.s)* |
|  | - | IU035200 | | Netherlands | *Vrn-A1b.6* | *vrn-B1 (VRN-B1.f)* |
|  | Chrysosperhum | UA0300219 | | Syria | *Vrn-A1b.6* | *vrn-B1 VRN-B1.f)* |
| *Triticum*  *turgidum* L. | Coles Selection | | CItr 13712 | USA | *Vrn-A1b.6* | *vrn-B1 (VRN-B1.f)* |
|  | R70 | | CItr 14445 | Ethiopia | *Vrn-A1c* | *vrn-B1 (VRN-B1.f)* |
|  | CI 7688 | | CItr 7688 | Russia | *Vrn-A1b.6* | *vrn-B1 (VRN-B1.f)* |
|  | H 33 A 12729 | | PI 191579 | Portugal | *Vrn-A1b.6* | *vrn-B1 (VRN-B1.f)* |
|  | Zerdakia | | PI 208912 | Iraq | *Vrn-A1i* | *vrn-B1 (VRN-B1.s)* |
|  | Seyed Zia | | PI 210372 | Iran | *vrn-A1b.3* | *vrn-B1 (VRN-B1.f)* |
|  | 1024-1 | | PI 211705 | Turkey | *Vrn-A1i* | *vrn-B1 (VRN-B1.f)* |
|  | Halcon | | PI 213571 | Argentina | *Vrn-A1b.1* | *vrn-B1 (VRN-B1.f)* |
|  | Gandum | | PI 220356 | Afghanistan | *Vrn-A1b.6* | *vrn-B1 (VRN-B1.f)* |
|  | Centigramun | | PI 221422 | Serbia | *Vrn-A1i* | *vrn-B1 (VRN-B1.f)* |
|  | Yellow Jarash | | PI 223173 | Jordan | *vrn-A1b.3* | *vrn-B1 (VRN-B1.s)* |
|  | Bufala Nera | | PI 264954 | Italy | *Vrn-A1b.1* | *vrn-B1 (VRN-B1.f)* |
|  | 504 | | PI 264991 | Greece | *vrn-A1* | *vrn-B1 (VRN-B1.f)* |
|  | 94 | | PI 266851 | UK | *vrn-A1b.3* | *vrn-B1 (VRN-B1.f)* |
|  | I-1-2274 | | PI 272496 | Hungary | *vrn-A1b.3* | *vrn-B1 (VRN-B1.f)* |
|  | Dalmatia 4 | | PI 278596 | Croatia | *vrn-A1b.3* | *vrn-B1 (VRN-B1.f)* |
|  | Dziwo | | PI 286075 | Poland | *vrn-A1b.3* | *vrn-B1 (VRN-B1.f)* |
|  | CI 3119 | | PI 28655 | Spain | *Vrn-A1c* | *vrn-B1 (VRN-B1.s)* |
|  | Blagunzi | | PI 295011 | Bulgaria | *vrn-A1b.3* | *vrn-B1 (VRN-B1.f)* |
|  | 2960 | | PI 306561 | Romania | *vrn-A1b.3* | *vrn-B1 (VRN-B1.f)* |
|  | 1584a | | PI 32039 | China | *Vrn-A1b.6* | *vrn-B1 (VRN-B1.f)* |
|  | - | | PI 323440 | Austria | *Vrn-A1b.1* | *vrn-B1 (VRN-B1.f)* |
|  | 412-IV/65 | | PI 345413 | BAG | *vrn-A1b.3* | *vrn-B1 (VRN-B1.s)* |
|  | Mauri | | PI 347135 | Afghanistan | *Vrn-A1i* | *vrn-B1 (VRN-B1.f)* |
|  | 778-VII/17 | | PI 350154 | Macedonia | *vrn-A1b.3* | *vrn-B1 (VRN-B1.f)* |
