# Supplementary material for: Novel alleles of the VERNALIZATION1 genes in wheat are associated with modulation of DNA curvature and flexibility in the promoter region
Source: BMC Plant Biol. 2016 Jan 27;16(Suppl 1):9. doi: 10.1186/s12870-015-0691-2 (PMC4895274; doi:10.1186/s12870-015-0691-2)
Supplement: Additional file 4: — “Effect of electrophoretic conditions on discrimination of VRN-A1 and VRN-B1 alleles”. “Optimization of PAGE for detection of the VRN-A1 and VRN-B1 alleles”. (DOC 1476 kb) [file 12870_2015_691_MOESM4_ESM.doc]

**Novel alleles of the *VERNALIZATION1* genes in wheat are associated with modulation of DNA curvature and flexibility in the promoter region**

**Additional file 4**

**Effect of electrophoretic conditions on discrimination of *VRN-A1* and *VRN-B1* alleles**

**Methods**

Amplification products were separated on 5 – 10% nondenaturing polyacrylamide gels (mono/bis-acrylamide ratio 29:1 – 82:1) in 0.5-1.38 TBE buffer (45-123 mM ionic strength), at temperatures 10-40 °C, with and without adding 15 mM Mg^2+^ ions, under 2.5-10 V/cm until the bands had migrated 50-70 % of the length of the gel.

**Results**

The low temperature, low ionic strength, and high concentration of acrylamide and Mg^2+^ ions are known to lead in the increase of anomalous slow migration of PCR fragments in polyacrylamide (PAA) gels. Nevertheless, it was found that not all of these conditions are suitable for clearly resolution of the *VRN-A1* and *VRN-B1* alleles identified in this study. While the adding of Mg^2+^ ions increased difference in migration rate between fragments indicating allelic variants of *VRN-A1* and promoter variants of *VRN-B1*, the time for electrophoresis was also significant increased (2-2.5 times). Furthermore, the results of PCR fragments separation for the *Vrn-A1b* variants in PAA gels with Mg^2+^ were poorly reproducible. Under low temperature (< 20°C) the 2-fold increase of electric field strength (from 5 to 10 V/cm) had not significant effect on the resolution of *VRN-A1* or *VRN-B1* variants. The room temperature (25-28°C in gel) the 2-fold decrease electric field strength (from 5 to 2.5 V/cm) leaded to significant retardation of anomalously slow migrating fragments and increase resolution for *VRN-A1* and *VRN-B1* variants, but it required up to two times longer run of electrophoresis.

Although low ionic strength (lower concentrations of gel buffer) lead to retardation of anomalously slow migrating DNA, the bands are diffused and blurred that making it difficult detection of difference between fragments with similar migration rate, such as amplicons of the *Vrn-A1b* variants. In contrast, the increasing of ionic strength (higher concentrations of gel buffer) the anomalous slow migration is suppressed. However a high ionic strength prevented diffusion of samples in gel, and leaded to sharper bands. It is important to note that the effect of ionic strength on anomalous migration DNA in PAA gels under low temperature (< 20°C) was insignificant (Fig. S2).

The significant effect had the structure and features of gel matrix. Particularly it was found that the increase of anomalous migration and electrophoretic resolution can be achieved if the electrophoresis rum in gels with higher mono/bis-acrylamide ratio (82:1 ratio was optimal). The increasing of acrylamide concentration to 10% accompanied by the increase of differences in the migration rate of PCR fragments at room temperature. However it had strong negative effect on bands resolution between *VRN-A1* and *VRN-B1* under lower temperature (Fig. S2). In addition, the high acrylamide concentration increased the time of the analysis, current through gels and, hence, temperature of gel during electrophoresis.

The greatest impact on the retardation of anomalous slow migration of PCR fragments and hence the increase of bands resolution in the *VRN-A1* and *VRN-B1* variants was the lower (Fig. S2). In general, a lower temperature requires for longer electrophoresis. However, since at low temperature (< 20°C) the increase of electric field and ionic strength had insignificant effect, and more concentrated polyacrylamide in gel had negative effect on the separation of the *VRN-A1* and *VRN-B1* amplicons the electrophoresis in lower percentage gels (6.6 %) with high ionic strength (123 mM) and under high electric field (5-10 V/cm) was run. In the result, the sharp bands with high resolution of PCR fragments for perfect detecting of *VRN-A1*and *VRN-B1* variants were obtained during relatively short time (1.5 - 3 h, depending on electric field strength and current).

**Figure S2.** The effect of polyacrylamide gel concentration, ionic strength and temperature on the change of distance between pair of PCR fragments of *VRN-A1* and *VRN-B1* alleles, measured relative distance between amplicons of *Vrn-A1e* (659 bp) and *vrn-B3a* (1768 bp) for which anomalous migration is not observed. Electrophoresis was run under 5 V/cm. The curve line indicates the difference in distance for corresponding pairs of PCR fragments after the conditions were optimized for electrophoresis at low (LT) and room (RT) temperature. The PCR fragments of the different *VRN-A1* and *VRN-B1* allelic variants were clearly resolved during PAGE separation regardless the temperature after optimization of electrophoretic conditions. M – DNA molecular size marker indicates 1000 and 1200 bp fragments for *VRN-B1* variants and 600, 700 and 800 bp for variants of *VRN-A1*; the marker bands migrate anomalously slow in PAA gels at given conditions.


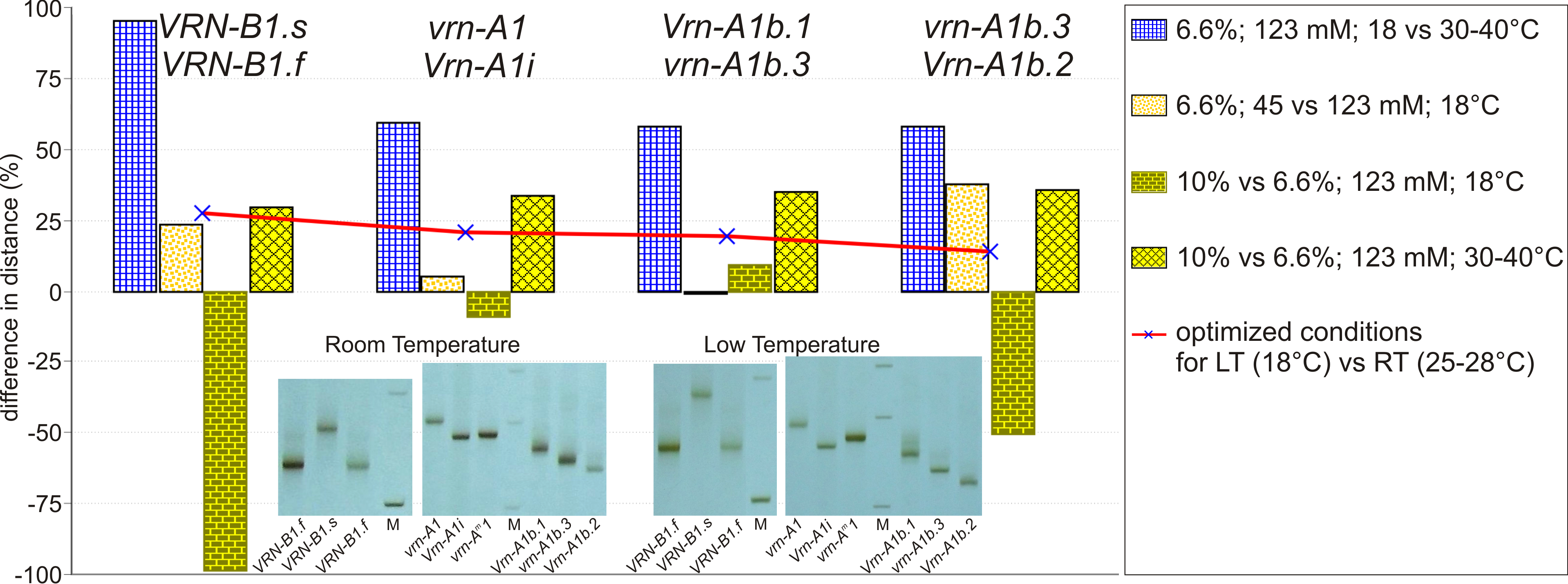


**Optimization of PAGE for detection of the *VRN-A1* and *VRN-B1* alleles**

The difference in curvature and flexibility of DNA molecules caused by the mutations within VRN-box of the identified *VRN-A1* allelic variants or deletions within A-tracts for promoter variants of *VRN-B1*. This leads to modulation in rate of anomalously migrating PCR fragments. Anomalous migration was observed only in PAA gels but not observed in agarose gels. Overall, the retardation of anomalously slow migrating PCR fragments and sharper bands are the especially important for identification of the *Vrn-A1b* variants, where *Vrn-A1b.1* / *Vrn-A1b.6* and *vrn-A1b.3* / *Vrn-A1b.2* show a small difference in migration rate, and also are the principal in discrimination variants of *VRN-B1*. The low temperature reduces the conformational dynamics and is a critical for the stabilization of shape of the DNA molecules, while high ionic strength results in sharper bands. The effect of Mg^2+^ ions on the migration of DNA fragments in PAA gels is known to be sequence dependent. Due to this and due to increasing duration of electrophoresis, it is not recommended to use magnesium ions during discrimination of the *Vrn-A1b* variants. This is primarily because the migration rate of PCR fragments will be affected by mutations outside of the VRN-box leading erroneous and poorly reproducible results. Unfortunately, failed to choose of electrophoretic conditions to unambiguously discriminate the *vrn-A1b.3* / *Vrn-A1b.5* and *vrn-A1b.4* / *Vrn-A1b.6*, which can be still identified by sequencing. On the other hand, *vrn-A1b.4* and *Vrn-A1b.5* were found in a single wheat accession only. Furthermore, under low temperature (10 °C) and high electric field strength (7.5-10 V/cm), the fragments of *Vrn-A1b.5* migrated slower than *vrn-A1b.3*, while *vrn-A1b.4* was slower than *Vrn-A1b.6*, although in both cases these differences were insignificant*.*

Based on revealed features of anomalous migration, the electrophoretic conditions were optimized to obtain the minimal loss of resolution (< 30 %) while electrophoresis run under low and room temperature (Fig. S2). Thus, the electrophoresis in 6.6% polyacrylamide gels at mono/bis-acrylamide ratio 82:1, under low electric field (2.5 V/cm) and high ionic strength (123 mM, x1.38 TBE in gel) was found as the most suitable when PAGE runs at room temperature, while the electrophoresis at low temperature (10-20 °C) with high electric field strength (5-10 V/cm) is recommended for the best results and time preservation. Nevertheless, electric field more 5 V/cm not needed to be increased if current exceeds 20 mA.
